# Multidimensional Sustainability Assessment of Chemical Valorization Pathways for PET Waste: A Literature-Based Early-Design-Stage Index Including Technology Readiness

**DOI:** 10.1021/acsomega.6c02141

**Published:** 2026-08-14

**Authors:** Daniel Alberto Pulido-Peña, Luis López Rojas, Paulo César Narváez-Rincón

**Affiliations:** Grupo de Procesos Químicos y Bioquímicos, Departamento de Ingeniería Química y Ambiental, Universidad Nacional de Colombia, Carrera 30 45-03 - Edificio 412, Bogotá 111321, Colombia

## Abstract

Inadequate waste-management practices have led to significant environmental impacts, requiring the development of sustainable valorization alternatives. The exponential growth of plastic waste represents a major challenge that must be addressed through coordinated global strategies. Among polymers, polyethylene terephthalate (PET) is one of the most produced and used worldwide, mainly for food and beverage packaging. It offers significant opportunities for the implementation of circular economy practices due to its inherent properties. Chemical processes can effectively return PET to its original components by molecular transformation, facilitating its reintegration into the production cycle. This study established a sustainability assessment for the chemical valorization of PET waste, incorporating technological, economic, environmental, and social dimensions with their corresponding indicators, all based on data compiled from a comprehensive literature review. The technological dimension was defined to allow the decision-maker to compare the technological readiness level (TRL) of each pathway. A Sustainability Index (SI), using selected indicators, was developed and applied to evaluate seven chemical valorization pathways. The sustainability assessment identified the top three alternatives in a base case: glycolysis as the most sustainable pathway (SI = 0.20), followed by basic hydrolysis (∼0.22) and microbiological degradation (∼0.25). Additionally, a sensitivity analysis was conducted by varying the level of importance of the dimensions, revealing the most influential factors for each valorization pathway. These findings underscore the potential of these alternatives to address the complex challenge of PET waste management, offering more sustainable solutions compared to conventional practices such as landfilling or incineration.

## Graphical abstract

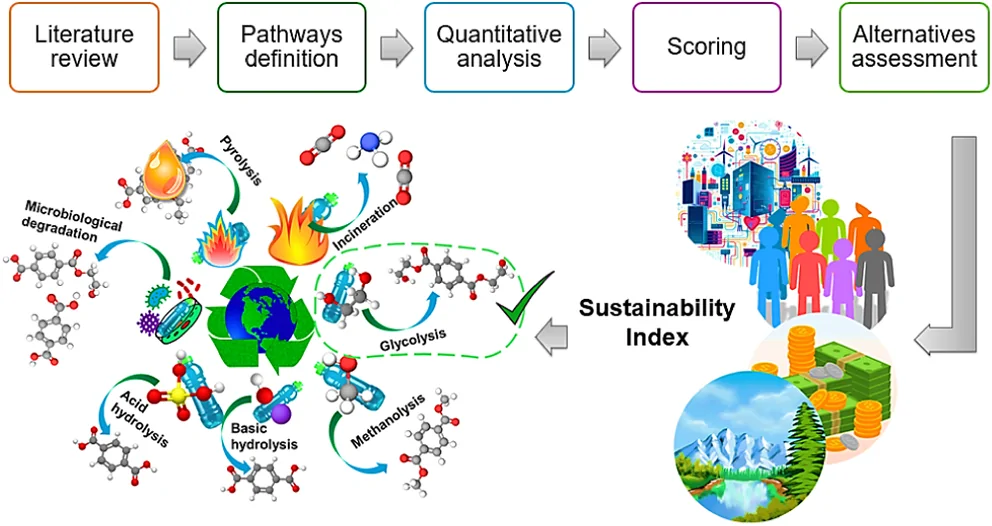

## Introduction

Plastics are a family of formulated materials composed essentially of polymers. Due to the wide range of properties, performance, and applications, as well as the economic advantages compared to other materials, plastics are present in multiple products required in modern life. Polymers are the main constituent of plastics conferring them most of their properties. Currently, most polymers are derived from petrochemical feedstocks obtained from crude oil and natural gas. Plastics are commonly classified according to the chemical structure of their polymer backbone (e.g., olefinic, phenolic, amino, polyester, etc.).^1^ However, formulations generally include additives, such as organic compounds, organometallics, or inorganic materials, which improve polymer performance or provide specific functionalities, broadening the potential applications and market opportunities for plastics. Among the polymer additives, plasticizers which increase the flexibility and workability, pigments that add colors to the plastic, lubricants used for making them more fluid, stabilizers which prevent degradation by UV light or oxidation, flame retardants that inhibit ignition of the plastic and reduce heat release while combusting, and impact modifiers which improve the toughness and durability against impacts, are the most commonly used.^2^

The increasing production and use of plastics have led to a significant rise in plastic waste generation. According to historical data from 1950 to 2017, approximately 7 billion tons of the 9.2 billion tons of plastics produced worldwide have become waste.^3^ In addition, about 23% of mismanaged plastic waste has been disposed of primarily in landfills,^4^ which is considered an unsustainable option due to the extremely slow degradation rate of plastics, requiring decades to thousands of years to degrade 50% of their mass.^5^ Furthermore, plastic waste has low density and occupies significant volume, exacerbating the limited capacity of landfills, which are increasingly burdened by growing waste streams. Over the last two decades, global plastic production has increased dramatically, accompanied by a parallel rise in plastic waste generation. In 2008, worldwide plastic production was estimated at 245 million tons, with polyethylene (PE) as the dominant resin, while polyethylene terephthalate (PET) accounted for approximately 20% of thermoplastics.^4^ This growth led to significant waste generation, reaching an estimated 141 million tons in 2015, corresponding to 37% of the total plastic produced that year.^6^ Production continued to increase in subsequent years, rising from 400 million tons in 2016 to 441 million tons in 2018.^7^ This upward trend was further intensified during the SARS-CoV-2 pandemic, as changes in consumption patterns led to increased demand for plastic products.^8–10,11^ In 2020, Houssini et al. reported that, of the 362 million tons of plastics manufactured worldwide, only 10.5% of the resulting waste was recycled, while 28.5% was landfilled and 24.9% was incinerated. Additionally, approximately 29.6 million tons (8.2%) of plastic waste were mismanaged, allowing dispersion into terrestrial and aquatic environments.^12^ Consequently, it is estimated that at least 2.29 million tons of waste entered the oceans in that year alone.^13,14^

Projections indicate that between 2020 to 2040, plastic leakage into the environment could increase by up to 50%, reaching approximately 30 million tons.^6^ This trend is further exacerbated by the presence of microplastics, which constitute an additional and growing environmental threat. Due to their small size, microplastics can accumulate across various elements of the biosphere, including the atmosphere, and subsequently enter food chains, ultimately leading to human exposure. Scientific evidence suggests that microplastics can disrupt the intestinal microbiota and induce inflammatory responses in the digestive system, as well as potentially cause toxic effects on the circulatory system and oxidative stress in the lungs when inhaled.^15^ Furthermore, their persistence has contributed to an estimated accumulation of up to 300 million tons of plastics in aquatic environments, doubling the number of plastics on the seabed projected for 2020.^6^

As previously mentioned, polyethylene terephthalate (PET) is one of the most widely used plastic materials. It is a polyester produced via polycondensation reactions, either by esterification of terephthalic acid with ethylene glycol or by transesterification of dimethyl terephthalate with the same glycol.^16^ PET exhibits desirable properties such as transparency, low weight, good barrier performance, and UV resistance, making it suitable for a wide range of applications, particularly in food packaging, beverage bottles, films, and textile fibers.^16,17^ PET is highly recyclable and is identified with recycling code 1 (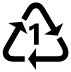), owing to its ability to retain quality over multiple recycling cycles and its versatility.^18^ Conventional recycling of PET involves shredding, cleaning, and reprocessing into flakes, pellets, or sheets.^16^ In some cases, PET waste is also used for energy recovery, generating heat and/or electricity depending on the use of combustion gases.^16^ Bottle recovery represents the primary source of recycled PET, accounting for 96% of the total collected, due to the maturity of collection and processing technologies.^19^ In the United States, PET bottle recycling rates increased from 28.6% in 2021 to 30.2% in 2024.^18,20^ In Europe, a significant share of recovered PET is downcycled into lower-value products; for example, in 2022, only 31% of the 1.8 million tons recovered was used for bottle production, while 69% was allocated to applications such as trays and textile fibers.^19^ By 2023, the installed PET recycling input capacity in Europe reached 3.2 million tons.^21^

PET can be revalorized through multiple pathways, some of which are currently in use.^22^ These are commonly classified into four categories based on the processing mechanism. Primary recycling (re-extrusion) involves the on-site processing of clean, homogeneous PET waste for reuse in similar applications.^22^ Secondary recycling (mechanical) includes the recovery, cleaning, and reprocessing of plastic waste into pellets for further use in other applications; however, repeated processing can decrease molecular weight and, consequently, reduce the material’s physical and mechanical properties.^16,22^ Tertiary recycling (chemical) requires the use of chemical agents to depolymerize PET through reactions such as hydrolysis, methanolysis, glycolysis, aminolysis, and ammonolysis. The first three processes enable the recovery of monomers suitable for repolymerization into high-quality PET.^16,22^ Quaternary recycling (energy recovery) makes use of the energy content of plastic waste through incineration, although its application is undesirable due to the potential release of pollutants and associated environmental impacts.^22^

Due to its recyclability, PET represents a key material for advancing sustainable waste management, in line with United Nations (UN) directives that promote waste reduction, reuse, increased recycling rates, and the use of recycled plastics.^23^ These actions align with Sustainable Development Goals (SDGs) 12, 16, and 17, supporting both policy development and industry transformation within a broader societal context.^3^ PET recycling also contributes to the transition toward a circular economy by promoting resource efficiency and waste minimization.^23^ In this context, PET recycling actions contribute to the principles of the Triple Bottom Line (TBL) framework, generating value for people, planet, and profit, represented in environmental, social, and economic dimensions with the aim of guiding toward more sustainable decision-making.^24^ Therefore, evaluating the sustainability of PET recycling alternatives is crucial, as the industry has recognized sustainability as a critical factor for the long-term success and viability of new projects, especially in the context of global challenges such as climate change and social inequality.^25^

Different studies intended to select the most sustainable alternative for PET plastics. Amirudin et al.^17^ used the Analytic Hierarchy Process (AHP) integrating technical and context criteria to evaluate different PET bottle waste management technologies in Indonesia. Prioritizing the most suitable technologies for developing countries, pelletization (secondary type) resulted in the most suitable option to implement. Similarly, Al-Thani et al.,^26^ integrated TOPSIS-COV (Technique for Order Preference by Similarity to Ideal Solution- Covariance) methodology to select a sustainable PET waste management technology in the State of Qatar. In this study, recycling alternatives were evaluated based on their proximity to an ideal solution, adjusting the weights considering the correlation between criteria; as result, the best strategy was the so-called “Closed Loop”, a secondary type mechanical processing methodology. Foolmaun and Ramjeeawon^27^ aimed to identify the most sustainable alternative for disposal of used PET bottles in Mauritius, considering both environmental and social impacts conducting a Social Life Cycle Assessment (S-LCA) a methodology that evaluates the social and socioeconomic impacts of a product or process throughout its entire life cycle, resulting in a proposal of 75% reprocessed flakes and 25% landfilled combination, which represents a combination of secondary and primary types. Choudhary et al.^28^ assessed the economic and environmental impact of mechanical recycling of PET waste in India. The authors acknowledging the limitations of a study realized on a specific region, evaluated scenarios in which the power source for the machinery is linked to renewable and nonrenewable sources. A solar powered filament production for recycling PET resulted as the most suitable scenario combining the energy source and the mechanical process. Martin et al.^29^ performed a comparative life cycle assessment for traditional PET waste management options in Bauru (Brazil), evaluating mixed scenarios of landfilling, incineration, and mechanical recycling aiming to find the better alternative of disposal referring particularly to the environmental impact, finding that, recycling is a better option than incineration. Barjoveanu et al.^30^ evaluated the environmental and economic impact of manual selection, automated selection, and a mixture of both methods in the classification of PET trays. The objective was to decide if incorporating specific separation processes improves the environmental and economic performance of a materials recovery facility (MRF) in Molfetta (Italy), concluding that upgrading the sorting technology diminishes both environmental impacts and operational costs.

The aforementioned studies identified suitable solutions for PET waste management by comparing alternatives within the four recycling categories, assessing the application of an individual alternative or the hybrid integration between alternatives within the four categories in a specific geographical context pairing environmental metrics with either economic or social factors. However, there remains a gap in research that provides a truly holistic evaluation encompassing the entire sustainability triple bottom line. For that reason, this study proposes a sustainability index to identify the most sustainable alternative. This index is a numerical instrument that assesses in a quantitative way sustainability by integrating environmental, economic, and social dimensions, with the novelty of adding a technological dimension based on the Technology Readiness Level (TRL). In this work, Tertiary and Quaternary transformation processes are considered as alternatives given their potential to recover the polymer without compromising material’s quality.^16^ Furthermore, using an early design stage approach, the proposal aims to provide a baseline comparison of chemical pathways according to their intrinsic characteristics without considering local conditions. This enables the identification of the most favorable routes from a sustainability perspective and supports subsequent process design for PET waste treatment based on an informed selection of reaction pathways.

## Methodology

### General Description

Seven pathways for PET chemical valorization were identified using a state-of-the-art literature review with a proposed search equation. Then, a sustainability analysis was established following the general guidelines for sustainability assessment proposed by Narváez et al.,^31^ which implies the formulation of a set of principles to subsequently establish criteria required for guiding the selection of indicators in accordance. The principles used in the study are tied to the Triple Bottom Line framework focused on the analysis of chemical processes, including economic feasibility of process, security aspects related to the inherent hazards of chemical substances focusing on the minimization of risks of damaging both humans and the environment.^31^ For the selected indicators, the relevant information to calculate the corresponding values was extracted from the literature review conducted in preparation for a mathematical normalization process. After that, the normalized values were organized into a matrix for the subsequent calculation of the *Sustainability Index*. Then, a sensitivity analysis was conducted varying the level of importance of each considered dimension to observe the impact on the results. This approach serves as a route selection index, providing a basis for subsequent techno-economic and Life Cycle Assessment (LCA) to support the implementation of the most sustainable routes. The concepts and considerations for this process are presented in detail in the following sections.

### Literature Review and Information Treatment

Strategically, the starting point was the collection of information related to the chemical recycling processes of PET waste. Facing the current global plastic pollution problem, multiple technological alternatives have been developed to address the amount of residue produced at a worldwide scale, conceiving solutions aimed to valorize the residue to useful feedstocks either via conversion to raw materials or the production of energy.^32^ A search equation was proposed and used to find publications focused on the exploration of traditional approaches and emerging technologies for PET waste processing and the possible indicators suited for their sustainability evaluation in scientific journals databases such as Scopus and Google Scholar as presented in Table 1. Once the information was collected, the relevant data was categorized and organized into two groups, technologies and sustainability indicators.

**Table 1:** Considered Elements in the Search Strategy Used for the Collection of Information About Chemical Recycling of Polyethylene Terephthalate

Characteristic	Description
eq 1	Sustainability AND Polyethylene AND Terephthalate AND Processing
eq 2	PET AND Sus* AND Processing
Databases	Scopus and Google Scholar
Period explored	2010–2024
Filter	Title, abstract, keywords
Type of documents	Articles and reviews

The retrieved set of technologies and indicators underwent a screening process to establish the general set of indicators and technologies mentioned previously for the sustainability assessment, according to the following considerations:

- Variations of the same technology were reduced to their most general definition. Each technology was selected based on its fundamental chemical basis. Hence, standard reaction conditions and solvents are considered, discarding experimental or novel alternatives.
- Similarly, indicators were considered in their broadest definition without specifics, e.g., soil pollution → Terrestrial Ecotoxicity Potential (TEP), water pollution → Hazard to Aquatic Life (HAL).
- Indicators were discarded if they could not quantify at least one chemical substance in any of the pathways.
- Aspects related to the physical or geographical location in the definition of the indicators were grounded for exclusion, since a generic, location-independent specific case is being analyzed.
- Specific economic conditions of the technologies, external to their fundamental reactions, were not considered in the selection of indicators.
- Economic indicators such as CAPEX/OPEX (Capital Expenditure/Operating Expenses), energy or labor costs, and other indicators related to the operation of a plant were not considered because the study focuses on the specific evaluation of the reactions for their selection, and not on the planning and implementation of a process, all in an early design stage approach.

### Determination of Values of Selected Indicators Using Specific Chemical Data: Mass Balance, Operating Conditions, and Economic Aspects

For the quantification of the defined indicators, it is necessary to calculate the mass of each compound involved in the reactions for each technology assessed. This must be done to ensure proper scaling up for the degradation of an equivalent amount of PET. The mass balance was calculated by either increasing the PET-to-substance ratio or adjusting the reagent ratios as stated on the reactions involved. This calculation did not consider technical aspects of the design of a hypothetical reactor, nor any type of downstream treatment of the reaction product streams. For the balance, a stream of pure PET as well as catalyst and solvent (if required) are considered as inputs, and streams of main products, byproducts along with other reagents are considered as outputs, as presented in Figure 1.

**Figure 1:**
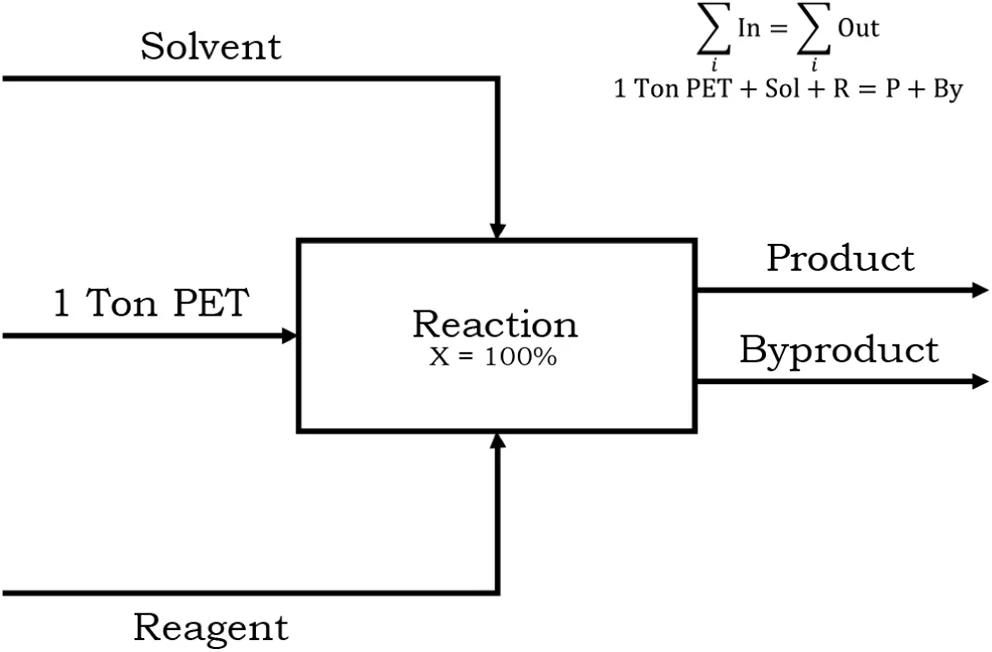
General process flow diagram (PFD) used for mass balance calculation.

Additionally, the following factors and assumptions were established:

- The calculation basis was 1 ton of PET, without additives or colorants.
- Reaction’s conversion is 100%.
- Particular solvents and catalysts are considered, when required.
- Possible subproducts not related to the degradation of plastic were included, for example, salts resulting from the neutralization or regeneration of a product of interest.

In the case of social indicators related to safety, the operating conditions and the identification hazards related to the chemical compounds involved in each pathway are required. For the latter, flammability and reactivity ratings were derived from the NFPA 704 standard and used as quantitative descriptors of safety-related information. Likewise, chemical substances were characterized in their inherent dangers of handling and disposal in accordance with what is described in the H statements (Hazard codes), which are part of the Globally Harmonized System (GHS) as a mechanism of immediate risk identification,^33^ the indicators were estimated under the normalized scale described by Narváez et al.,^31^ which considers the inherent hazards of manipulation of chemical substances to account the possible risks, safety and health impacts that may affect both the direct handlers of the substances and the general population residing near the point of use. For information regarding the economic aspect, commercial wholesale prices (in USD per ton) of each chemical product involved in a reaction were consulted in chemical market databases, accounting for standard industrial grades. The values for the indicators of the environmental dimension were based on those determined in Guinée’s Handbook,^34^ except for “Hazard to Aquatic Life” indicator which was determined by Narváez et al.^31^ Finally, the maturity of each alternative was the only indicator of the technological dimension, quantified through the maximum TRL value reported for each technology. All the data collected and used in the subsequent stages of this study were appropriately compiled and presented in the Supporting Information, for the sake of brief and concise reading of the relevant aspects in the present article.

### Normalization of Indicators

To obtain comparable information, it is required to condense the list of calculated indicators into a single numerical value for each dimension. However, this operation has a drawback related to the dimensional consistency.^35^ To facilitate comparison among indicators, data normalization is required to obtain a dimensionless set of values while preserving each indicator's meaning and behavior. Thus, a range from zero (0) to one (1) is defined for each indicator on a dimensionless scale, where the values close to or equal to zero represent the most sustainable alternative, and those close to or equal to one would be interpreted as less sustainable. The normalization method was applied on the rationale underlying each indicator, as defined by its conceptual definition. This aspect is relevant because all indicators do not follow the same logic in their numerical interpretation, since lower values do not necessarily indicate a benefit, but may instead represent a penalty or adverse outcome. In such cases, the normalized value may be inverted to ensure consistency in the directionality of the scale.^35^ To ensure a well-distributed set of values and better highlight the impact of each alternative on the indicators, several normalization methods were evaluated for each indicator, and the one providing the most consistent and meaningful distribution was selected. The selected methods tested were:

1 $$x=\frac{x_{i}}{\max x_{i}}$$

2 $$x=\frac{x_{i}-\min\left(x_{i}\right)}{\max\left(x_{i}\right)-\min\left(x_{i}\right)}$$

3 $$x=\frac{x_{i}}{\underset{i}{\sum }x_{i}}$$

4 $$x=\frac{x_{i}}{\sqrt{\underset{i}{\sum }{x_{i}}^{2}}}$$

Where *x* is the final normalized value and *x*_*i*_ are the obtained values of all alternatives for the indicator (*i*). A normalization method was considered appropriate if the normalized values preserved the overall trend and relative ordering of the raw data. This was verified by a strong monotonic or linear correlation between raw and normalized data sets, ensuring that the normalization process maintained the inherent structure of the data while enabling comparability across indicators. The detailed selection could be found in Supporting Information (Figure S1).

### Weight Assignment and Sustainability Index

The proposed analysis was based on a sustainability index that considers an aggregate value of all indicators within each dimension. First, $D_{i}^{\left(l\right)}$ represents the value of dimension *i*, calculated as the sum of its normalized indicators ${\mathrm{x̃}}_{i,j}^{\left(l\right)}$ , where *j* indexes the indicators within that dimension, divided by *m*_*i*_ which denotes their total number. These indicators correspond to pathway *A* ^(*l*)^, the superscript *l* refers to the evaluated pathway:

5 $$D_{i}^{\left(l\right)}=\frac{1}{m_{i}}\overset{m_{i}}{\underset{j=1}{\sum }}{\mathrm{x̃}}_{i,j}^{\left(l\right)}$$

The relative importance of the weight assigned to each dimension, *w*_*i*_, represents a fraction of total sustainability, such that 0 ≤ *w*_*i*_ ≤ 1. To examine the impact of this parameter, two weighting schemes are defined. When a specific dimension *k* is designated as the primary dimension, its weight *w*_*k*_ is assigned to a value within the range 0.20 ≤ *w*_*k*_ ≤ 0.75. That range provides flexibility in the application of the SI by accounting for extreme cases in the relative importance of each considered dimension. The remaining dimensions share the residual weight equally, with the distribution determined by the total number of dimensions *n*:

6 $$w_{i}^{\left(k\right)}=\{\begin{array}{l} w_{k}\;if\;i=k\\ \frac{1-w_{k}}{n-1}\;if\;i\neq k \end{array}$$

When *i* = *k* the weight is only assigned, e.g., if the assigned value is 0.4, as this study considers four dimensions, the other three will have a weight value of 0.2. This distribution is done to isolate the impact of the selected dimension *k*, ensuring that the observed variations can be associated solely with that specific attribute without omitting the relative contributions of the remaining dimensions. For the base case, equal weighting is considered, where all dimensions contribute equally:

7 $$1=\overset{m_{i}}{\underset{i=1}{\sum }}w_{i}^{\left(k\right)}$$

The Sustainability Index (SI) for an evaluated pathway (*A* ^(*l*)^) will then be the sum of the dimension value multiplied by the assigned importance weights:

8 $$SI^{\left(l,k\right)}=\overset{n}{\underset{i=1}{\sum }}w_{i}^{\left(k\right)}D_{i}^{\left(l\right)}=\frac{w_{k}}{m_{i}}D_{k}^{\left(l\right)}+\overset{n}{\underset{\begin{array}{l} i=1\\ i\neq k \end{array}}{\sum }}\frac{1-w_{k}}{\left(n-1\right)m_{i}}D_{i}^{\left(l\right)}$$

## Results and Discussion

### Retrieved Information: Technologies for Chemical Valorization

As a product of the review, from the use of both search equations, a total of 11 (11) technologies for processing the PET residues were identified, based on either mechanical or chemical principles. These technologies range from simple disposition to exploitation and restoration into the utility chain. The selected alternatives were open fill, sanitary landfill, incineration, pelletizing, hydrolysis, glycolysis, methanolysis, pyrolysis, open loop, closed loop, and microbiological degradation, which are described briefly in Supporting Information (Table S1). From that group of alternatives, only technologies based on chemical processing were selected for detailed study. This is because chemical pathways have advantages over mechanical valorization, such as the possibility of obtaining a wide variety of products beyond reprocessed polymer.^36,37^ Moreover, this type of recycling can focus on the recovering of raw materials of the polymer to synthesize a new one with equal or even superior quality compared to the original.^36,37^ In the same way, Tertiary category techniques of recycling are aligned with the 9-R principles of circular economy, as they allow to *Reduce* (R_2_) and *Reuse* (R_3_) the discarded residue material.^36,37^

In addition, the incineration process was also considered as comparative reference as this is one of the most openly adopted pathways of disposal in the world.^36^ Despite not being listed in the tertiary category, microbiological degradation was also considered in this study as a chemical alternative because it involves molecular transformation by the action of microorganisms. This is a promising technology currently in development, with the potential to compete with other chemical processes. The selected alternatives are accurately described in Table 2.

**Table 2:** Selected Processes and Their Classification According to the Recycling Type Principle

Technology	Category	Definition	Refs
Hydrolysis	Tertiary	Slow depolymerization of the clean PET residue via reaction with water molecules in conditions of high pressures and temperatures. It can be done in acidic, neutral, or basic mediums for the obtention of terephthalic acid and ethylene glycol, for their posterior conversion into raw materials for other purposes.	^16,22^
Glycolysis	Tertiary	Fast and low-cost depolymerization of the clean PET residue, reacting with ethylene glycol to produce bis(2-Hydroxyethyl) terephthalate (BHET) and other terephthalic glycols that can be used in the manufacturing of diverse polymers such as unsaturated resins, polyurethane foams, or acrylic covers. The reaction can be done with the use of a catalyst, or a solvent, in supercritical conditions or assisted by microwave radiation.	^16,22^
Methanolysis	Tertiary	Use of methanol for the conversion of PET residue to dimethyl phthalate releasing ethylene glycol. The reaction is conducted under high pressures and temperatures, obtaining two monomers useful for the repolymerization of new PET. It is usually catalyzed with organometallic substances, especially acetate types.	^16,22^
Pyrolysis	Tertiary	Decomposition of plastic waste via the direct heat at elevated temperatures in an inert atmosphere, using heating assisted by plasma or microwave radiation. It produces different organic products such as fuel oils or valuable combustion gases.	^17,36^
Microbiological degradation	Tertiary	Chemical decomposition of the polymer assisted with bacterial or fungi species taking advantage of the enzymatic activity of the microorganism, which can be found in nature or be genetical modified.	^38^
Incineration	Quaternary	Combustion of plastic waste, using it as a source of fuel for the obtention of heat or as a means of thermal energy generation via water vaporization. Usually, it represents the last and most convenient alternative of waste disposal when the other means cannot be afforded. The gas streams generated by combustion are difficult to treat properly and represent a serious source of pollution.	^16,17,26^

Each process described above can be associated with the fundamental chemical reactions involved in the transformation stage. In this study, the characteristics considered for the selected processing pathways are the most efficient reported in the literature, including operating conditions, reagents, catalysts, and solvents involved. For instance, “Microbiological degradation” was represented with the use of the *Candida antarctica* strain, which can process PET as described by Castro et al.^39^ Referring to “Hydrolysis”, acid and basic options were analyzed separately. For the acid option, sulfuric acid at 98% wt. was selected as the catalyst because it is the most common substance used in this procedure.^16^ For basic hydrolysis, sodium hydroxide in solution 98% wt. is reported due to its efficiency close to 100% in the conversion of PET to terephthalic acid.^40^ Additionally, for the “Pyrolysis” reaction, a characterization of mass products of this process is required for further calculations. For that reason, the analysis on pyrolysis products of PET conducted by Dimitrov et al.^41^ along with the results reported by Castro et al.^39^ were used for the corresponding mass balance of the process.

Except for “Glycolysis” and “Methanolysis”, the most usual reaction media for these reactions is water.^39,40^ For glycolysis, ethylene glycol is commonly used as solvent, whereas dichloromethane can be used in methanolysis to facilitate the subsequent products separation; both reactions can be catalyzed with zinc diacetate.^16,40^ A summary of the chemical pathways representing the selected processes is presented below:

Incineration:^32^ 
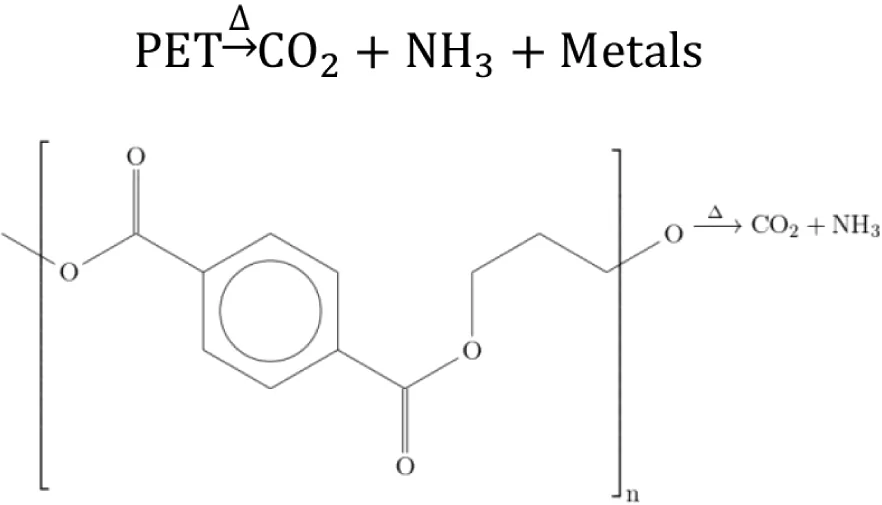


Acid hydrolysis:^16^ 
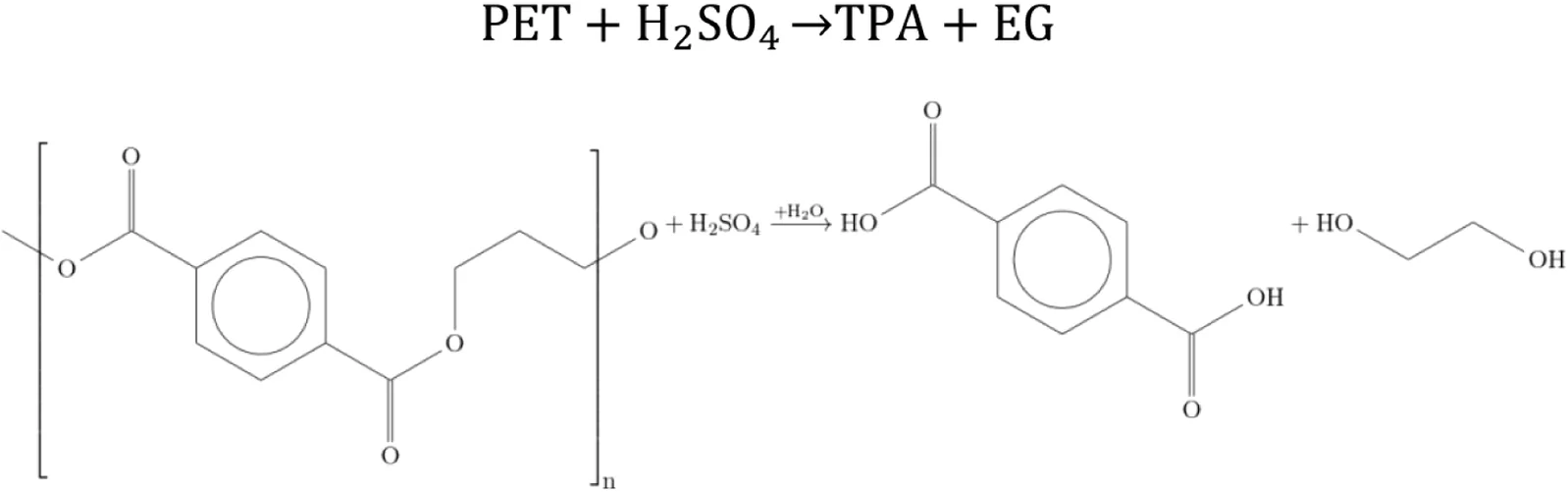


Basic hydrolysis:^16^ 
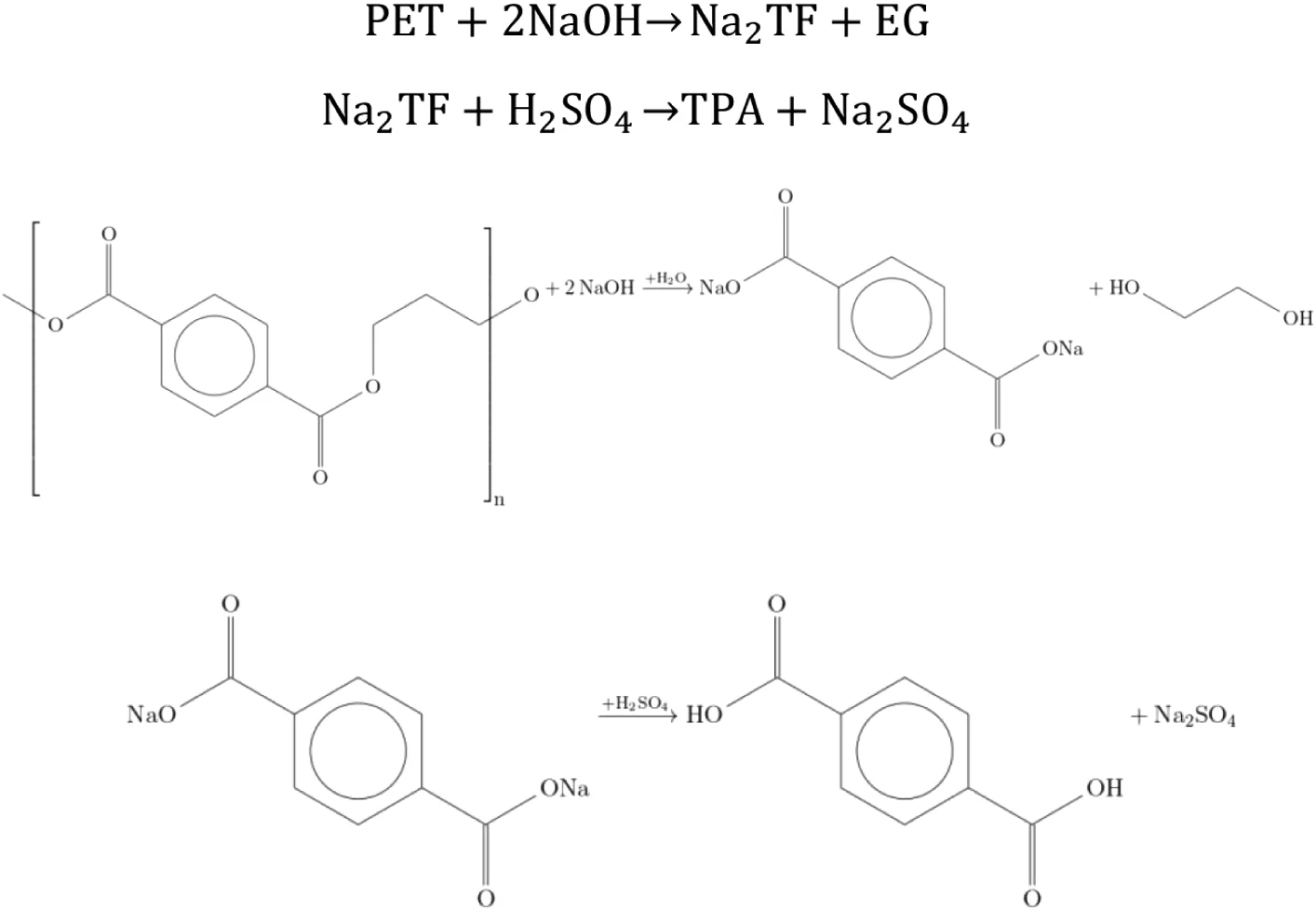


Glycolysis:^16^ 
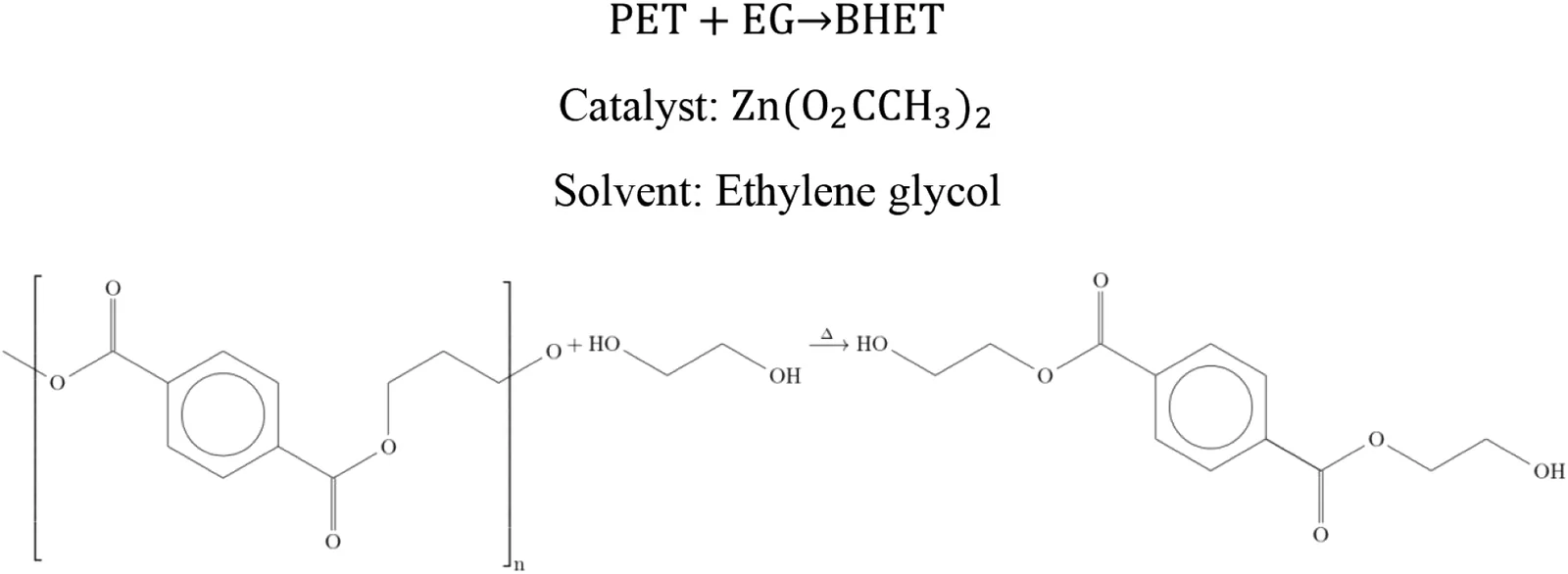


Methanolysis:^16^ 
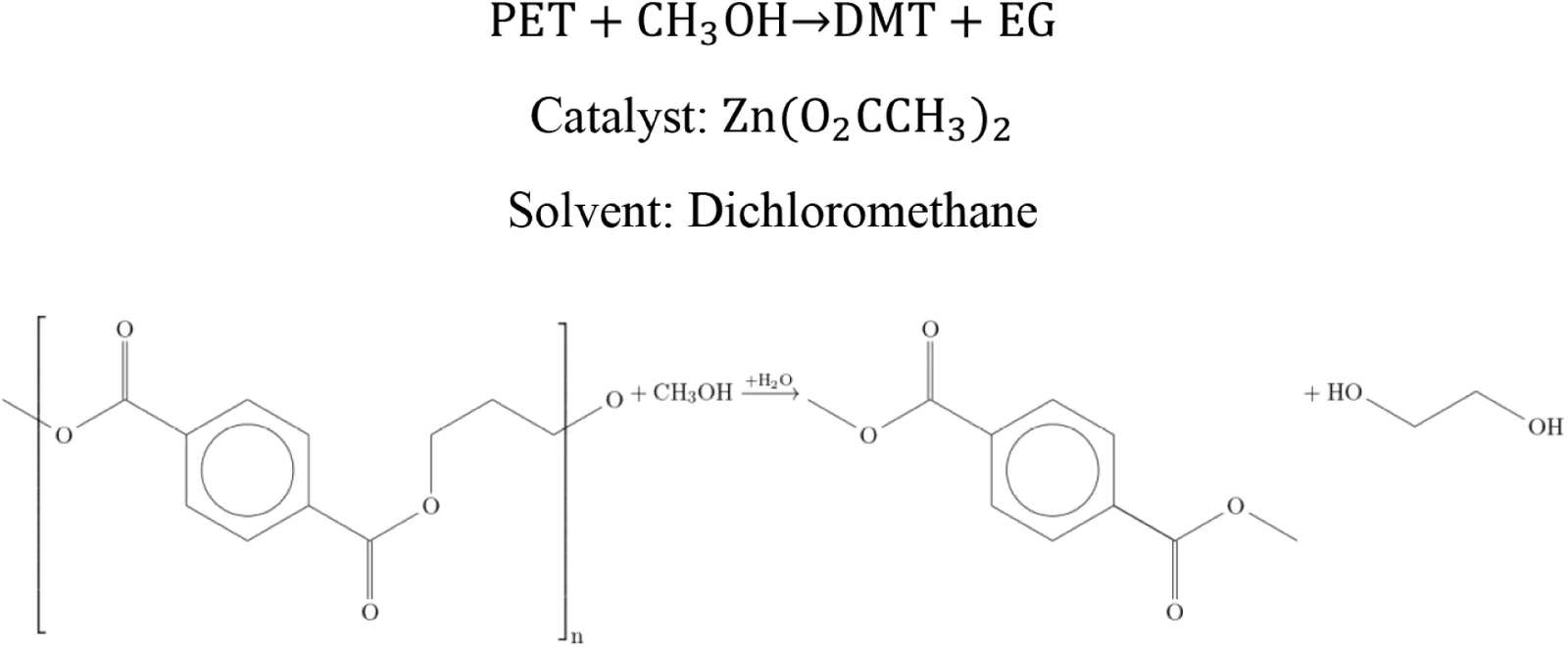


Pyrolysis:^41^ 
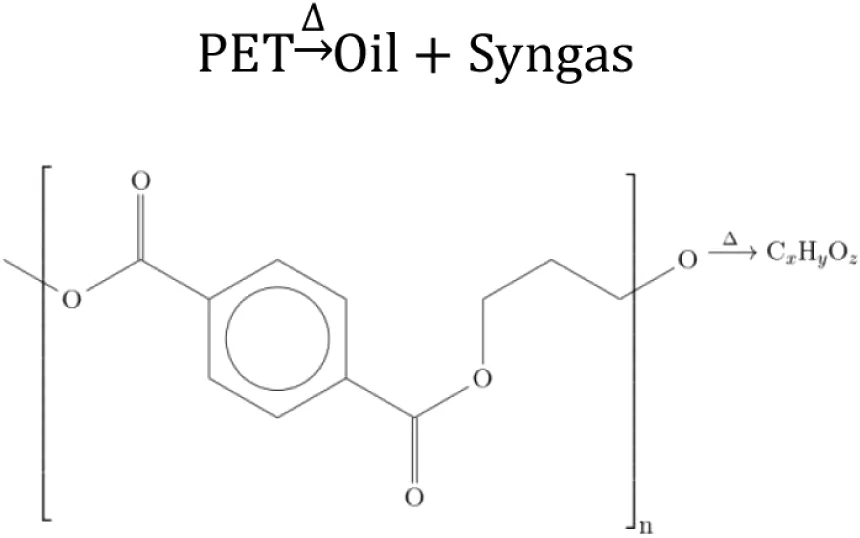


Microbiological degradation:^39^ 
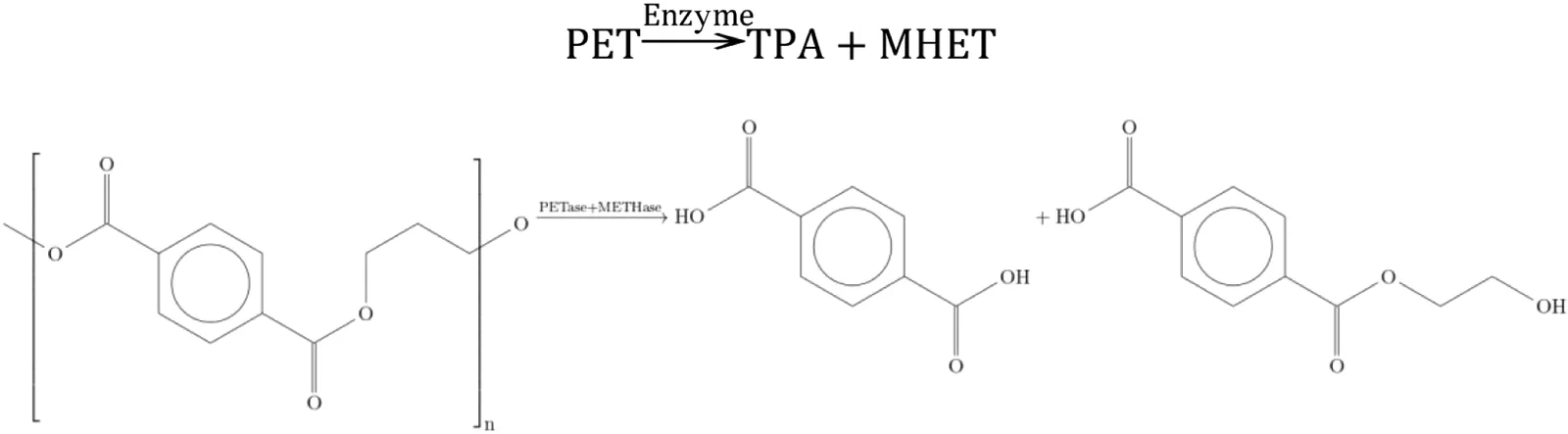


Where TPA: Terephthalic acid, EG: Ethylene glycol, Na_2_TF: Disodium terephthalate, Na_2_SO_4_: Sodium sulfate, BHET: Bis­(2-hydroxyethyl) terephthalate, CH_3_OH: Methanol, DMT: Dimethyl terephthalate, Zn­(O_2_CCH_3_)_2_: Zinc di­(acetate), and MHET: 2-Hydroxyethyl terephthalic acid.

### Retrieved Information: Definition of Sustainability Indicators

Similar to the identification of technologies, a total of forty-eight (48) sustainability indicators were identified through the literature review and are listed in the Supporting Information (Table S2). These indicators were subsequently subjected to a filtering process based on the clarity of their definitions and the feasibility of the data required for their calculation. As a result, a refined set of indicators was established to evaluate the selected chemical pathways from a sustainability perspective. This screening process excluded indicators based on the intrinsic properties of the chemical substances involved, as well as those requiring the specification of operating conditions or location-dependent parameters (e.g., country, city, or site) to accurately estimate their values.^17,26^ As previously established, the focus of this study is primarily on the reactions in an early design stage approach, rather than on the design or implementation of a complete recycling process.

Location-dependent exclusion was also applied to indicators associated with pollution in specific environmental compartments, such as water, air, or soil, within a defined geographic area. These indicators are often influenced by national or regional political and regulatory frameworks, region-specific productivity data used as a basis for estimations, and socioeconomic biases rooted in local market conditions and prevailing price structures. Additionally, indicators requiring demographic data in combination with direct stakeholder engagement, such as polls, interviews, or surveys, were also excluded from the final selection.^27^

Once the group of indicators collected from literature review were reduced, additional indicators related to the early stages of chemical process design were added to incorporate concepts of social and environmental security regarding the manipulation of chemical products and risks associated with their processing. Several of these metrics, formerly proposed by Narváez et al.,^31^ were selected to strengthen those sustainability dimensions. As result, the final list of the selected indicators for this study is presented in Table 3.

**Table 3:** Selected Sustainability Indicators for the Sustainability Assessment of the Selected Alternatives for Chemical Valorization of PET

Dimension	Indicator	Definition	Units	Refs
Environmental	Abiotic Depletion Potential (ADP)	Diminution of natural availability of metals as result of its mining extraction.	kg Sb eq.^a^	^34^
	Eutrophication Potential (EP)	Characterizes the possible environmental impacts related to the increasing of micronutrients.	kg PO_4_^3–^ eq.^b^	^34^
	Global Warming Potential(GWP)	Compares the absorption of infrared radiation per unit mass of a substance during its lifetime relative to CO_2_.	-	^31^
	Photochemical Oxidant Formation Potential (POFP)	Comparative rate of reaction with the OH* radical per unit mass of a substance regarding ethylene.	-	^31^
	Terrestrial Ecotoxicity Potential (TEP)	Referring to the potential damage that the release of toxic substances in the soil can cause to the organisms that inhabit it.	kg PDCB eq.^c^	^26,34^
	Hazard to aquatic life (HAL)	It refers to the short-term damage caused to marine organisms by exposure to toxic substances released in bodies of water.	-	^31^
Economic	Cost	Gross price of the raw material involved in the reaction pathway, including the catalyst.	USD	-
	Revenue	Gross sales price of the products and byproducts of the reaction pathway.	USD	-
Social	Carcinogenicity (Car)	Quantify the risk associated with the development of carcinogenic anomalies due to the manipulation of a chemical substance.	-	^31^
	Mutagenicity (Mut)	Quantify the risk associated with the development of mutations due to the manipulation of a chemical substance.	-	^31^
	Acute toxicity (AT)	Quantifies the risk of toxic effects associated with the consumption of a chemical substance.	-	^31^
	Flammability (F)	Referring to the risk associated with the temperature value at which a substance (solid, liquid, gas) is capable of combusting.	-	^31^
	Reactivity (R)	It quantifies the risk of an unstable substance that is capable of reacting with itself in a violent and exothermic manner, even in the absence of oxygen.	-	^31^
	Reaction temperature (RT)	It represents the human risk associated with the cooling or heating that a process experiences in its reaction stage.	°C	^31^
	Reaction pressure (RP)	It represents the human risk associated with the pressurization or vacuum that a process experiences in its reaction stage.	bar	^31^
Technological	Maturity	Describes the state of readiness of a technology.	-	-

a Sb: Antimony.

b PO_4_^3–^: Phosphate ion.

c PDCB: 1,4-Dichlorobenzene.

As previously mentioned, this study includes an additional dimension, the Technological, with the purpose of including process maturity, a critical aspect that stakeholders consider in the decision-making for a possible implementation. In this case, the Technological Readiness Level (TRL) was selected as indicator of the state of development of each alternative, measuring the availability of the technology for use in the near future. Technologies at lower TRL levels typically require longer development times to reach maturity required for the industrial implementation in chemical processing.

### Retrieved Information: Specific Chemical Data, Mass Balance, Operating Conditions, and Economic Aspects

In accordance with the considerations described in the methodology, all information collected and used for the mass balance calculations as well as the technical data related to the operating conditions of the recycling pathways, properties related to chemical hazards of the substances, and economic data on market purchase and selling prices, were compiled. That information is available in the Supporting Information provided (Table S3, Table S4, and Table S5).

### Indicator Values

Once the indicator values for each substance in a specific pathway were estimated, the sustainability impact of each alternative was calculated by taking the mass-weighted sum of the indicator values for all substances involved in that chemical pathway. Table 4 summarizes the calculation method associated with each indicator. Likewise, the normalization methods applied to each indicator are also presented, their selection was based on the data distribution observed after initial calculations.

**Table 4:** Mathematical Formulation and Normalization Methods of the Selected Indicators for the Sustainability Assessment Chemical Valorization Alternatives of PET^e^

Dimension	Indicator	Equation^b^	Normalization method^c^	Indicator range	Reference
Environmental	ADP (kg Sb eq.)	$\mathrm{ADP}=\underset{i}{\sum }{\mathrm{ADP}}_{i}\times m_{i}$	$x=\frac{x_{i}}{\max\left(x_{i}\right)}$	0≤x<∞	^34^
	EP (kg PO_4_^3–^ eq.)	$\mathrm{EP}=\underset{i}{\sum }{\mathrm{EP}}_{i}\times m_{i}$			^34^
	GWP	$\mathrm{GWP}=\underset{i}{\sum }{\mathrm{GWP}}_{i}\times m_{i}$			^34^
	POFP	$\mathrm{POFP}=\underset{i}{\sum }{\mathrm{POFP}}_{i}\times m_{i}$			^34^
	TEP^a^ (kg PDCB eq.)	$\mathrm{TEP}=\underset{i}{\sum }\underset{ecom}{\sum }{\mathrm{TEP}}_{ecom,i}\times m_{ecom,i}$			^34^
	HAL	$$\mathrm{HAL}=\underset{i}{\sum }{\mathrm{HAL}}_{i},{\mathrm{HAL}}_{i}=\{\begin{array}{l} 1.0,\;if\;H400\\ 0.75,\;if\;H401\\ 0.05,\;if\;H402 \end{array}$$		0≤x≤1	^31^
Economical	Cost (USD)	$\mathrm{Cost}=\underset{i}{\sum }{\mathrm{Cost}}_{i}\times {\left(1+r\right)}^{n}\times m_{i}$			
	Revenue (USD)	$\mathrm{Revenue}=\underset{i}{\sum }{\mathrm{Revenue}}_{i}\times {\left(1+r\right)}^{n}\times m_{i}$	$x=1-\frac{x_{i}}{\max\left(x_{i}\right)}$		
Social	Car	$$\mathrm{Car}=\underset{i}{\sum }{\mathrm{Car}}_{i},{\mathrm{Car}}_{i}=\{\begin{array}{l} 1.0,if\;H350\\ 0.50,if\;H351 \end{array}$$	$x=\frac{x_{i}}{\max\left(x_{i}\right)}$		^31^
	Mut	$$\mathrm{Mut}=\underset{i}{\sum }{\mathrm{Mut}}_{i},{\mathrm{Mut}}_{i}=\{\begin{array}{l} 1.0,\;if\;H340\\ 0.50,\;if\;H341 \end{array}$$			^31^
	AT	$$\mathrm{AT}=\underset{i}{\sum }{\mathrm{AT}}_{i},{\mathrm{AT}}_{i}=\{\begin{array}{l} 1.0,\;if\;H300/H310/H330\\ 0.75,\;if\;H301/H311/H331\\ 0.50,\;if\;H302/H312/H332\\ 0.25,\;if\;H303/H313/H333 \end{array}$$			^31^
	F	$F=\underset{i}{\sum }\mathrm{NFPA}:F_{i}$	$x=\frac{x_{i}-\min\left(x_{i}\right)}{\max\left(x_{i}\right)-\min\left(x_{i}\right)}$	0≤x<∞	
	R	$R=\underset{i}{\sum }\mathrm{NFPA}:R_{i}$			
	RT (°C)	$\mathrm{RT}=\frac{\underset{i}{\sum }{\mathrm{RT}}_{i}}{2}$			
	RP (bar)	$\mathrm{RP}=\frac{\underset{i}{\sum }{\mathrm{RP}}_{i}}{2}$	$x=1-\frac{x_{i}-\min\left(x_{i}\right)}{\max\left(x_{i}\right)-\min\left(x_{i}\right)}$		
Technological^d^	Maturity	TRL	$x=1-\frac{x_{i}}{9}$	0≤x≤9	

a Estimated under the assumption of an industrial ground.^34^

b For Microbiological degradation, the values were scaled up from the reaction taking place in 14 days.

c Retrieved from ref 35

d Considering the definition of TRL.

e *m*_*i*_/*m*_*ecom*,*i*_ is the mass of a particular substance. *r* represents the average dollar inflation rate up to 2026. represents the average dollar inflation rate up to 2026. *n* represents the average dollar inflation rate up to 2026. *H*### refers to a particular H-statement.

Following the calculation and normalization of the indicators, the data required for the sustainability assessment were organized into a matrix, presented in Table 5. This table has dimensionless values that facilitate the subsequent analysis of impacts by dimension of each chemical pathway considered. The corresponding raw data are available in Table S8 of Supporting Information.

**Table 5:** Normalized Indicator Matrix for the Sustainability Assessment of the Selected Alternatives for Chemical Valorization of PET

Alternatives								
Dimension	Indicator	Incineration	Acid hydrolysis	Basic hydrolysis	Glycolysis	Methanolysis	Pyrolysis	Microbiological Degradation
Environmental	ADP	1.09 × 10^–5^	0.14	1.00	1.96 × 10^–3^	2.14 × 10^–3^	0.00	0.00
	EP	3.13 × 10^–5^	0.03	0.03	0.14	1.00	0.11	0.08
	GWP	3.20 × 10^–3^	0.01	0.01	0.18	1.00	0.06	0.00
	POFP	0.00	0.02	0.02	0.24	1.00	0.08	0.00
	TEP	3.98 × 10^–4^	0.00	0.00	0.00	1.00	6.60 × 10^–5^	0.00
	HAL	1.00	0.00	0.00	0.26	0.32	0.26	0.00
Economical	Cost	0.02	0.04	0.34	0.08	1.00	0.07	0.04
	Revenue	0.95	0.95	0.00	0.94	0.79	0.89	0.95
Social	Car	0.50	0.00	0.00	0.00	0.25	1.00	0.00
	Mut	0.33	0.00	0.00	0.00	0.33	1.00	0.00
	AT	1.00	0.15	0.15	0.31	0.69	0.31	0.15
	F	0.00	0.09	0.09	0.18	0.55	1.00	0.00
	R	0.00	0.67	1.00	0.33	0.33	0.67	0.00
	RT	1.00	0.17	0.25	0.22	0.25	0.57	0.00
	RP	0.00	0.62	0.62	0.00	1.00	0.00	0.00
Technological	Maturity	0.00	0.33	0.33	0.00	0.11	0.00	0.33

As previously mentioned, the values closest to zero (0) correspond to a more sustainable performance. A notorious trend of benefit can be observed in Table 5 for the “Microbial degradation” alternative, which displays a higher frequency of zero-valued indicators than the other alternatives. In principle, biochemical processes of this type usually do not require chemical substances or mixtures with high environmental or social safety impacts. These processes employ microorganisms to be conducted; thus, strict care and monitoring is required over the operating conditions to ensure proper functioning, avoiding contaminating or toxic substances, in addition to generally operating at relatively low temperatures and pressures close to atmospheric pressure.^42^

The inclusion of a microbiological alternative enables the assessment of its potential advantages over the use of purely chemical pathways, especially regarding safety aspects related to chemical risks and the use of biological agents. However, it is worth mentioning that the variety of microbiological species identified for PET degradation gives rise to a wide range of conditions and possibilities that go beyond the scope of this study.^38^ For that reason, future studies should focus on a detailed evaluation of microbiological alternatives. This would enable a more rigorous assessment of their viability and feasibility compared to chemical processing strategies.

### Sustainability Index

A sustainability index was developed to analyze the pathways described above. This proposed index evaluates impacts across different dimensions, enabling an assessment of each pathway from a sustainability perspective. The resulting information can be used in early design stages to guide the selection of alternatives, as it allows decision-makers to compare their potential implementation in real-world conditions.

### Base Case and Index Sensitivity Analysis

An analysis of the sustainability index results for the base case, assuming equal weighting for all dimensions, shows that, except for methanolysis, the alternatives exhibit similar scores with a narrow distribution, as illustrated in Figure 2. Glycolysis stands out as the most sustainable alternative, with an index value close to 0.20, followed by microbiological degradation (∼0.22) and basic hydrolysis (∼0.25). All these alternatives exhibit lower index values than incineration (∼0.26).

**Figure 2:**
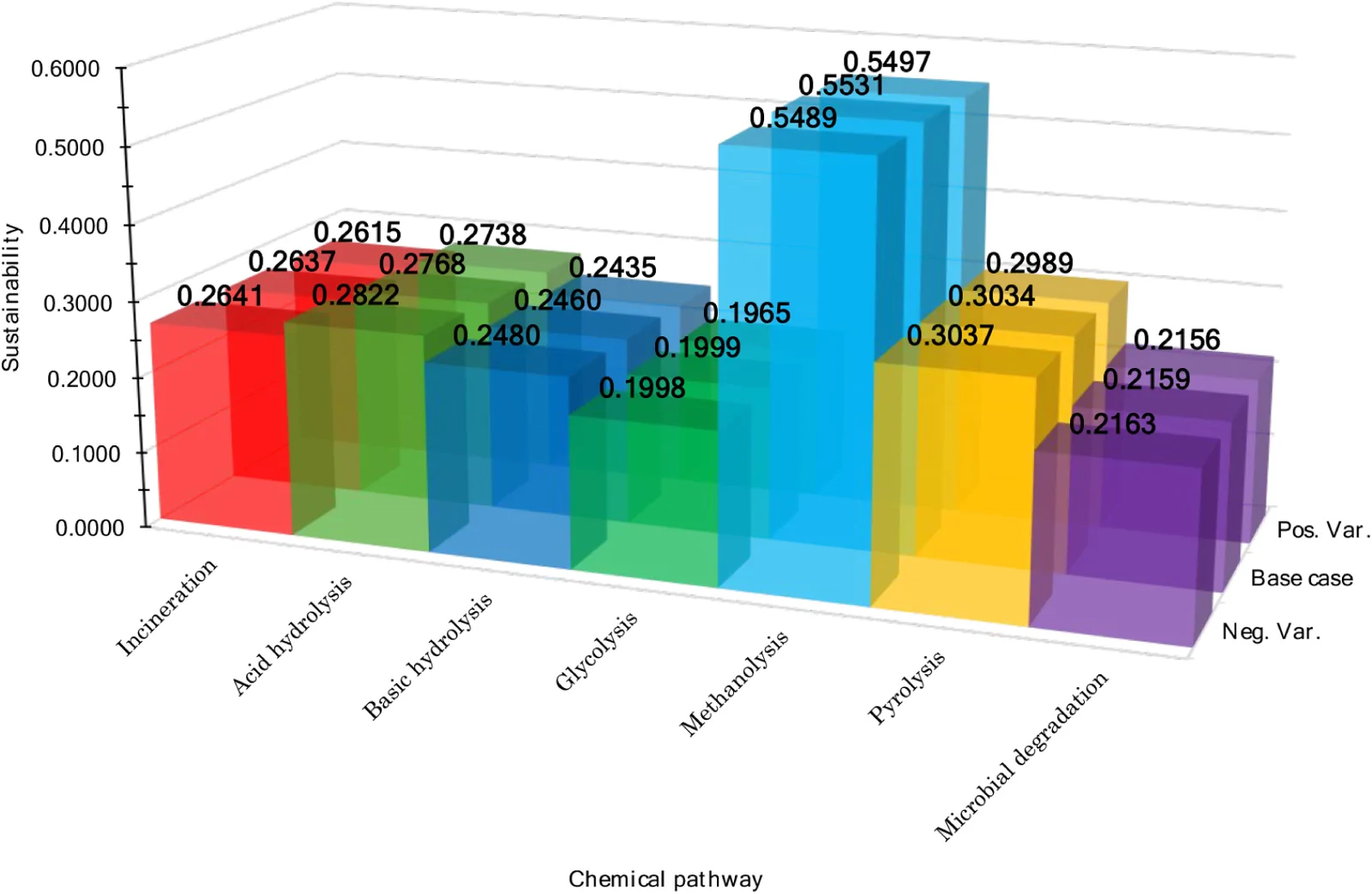
Sustainability index values for the selected alternatives for PET chemical valorization under the base case with equal weighting across all dimensions.

Acid hydrolysis achieved a sustainability index of approximately 0.28, nearly matching the top three alternatives. Given this proximity, it is relevant to assess whether variations in the index inputs could significantly affect this result. Therefore, it was decided to perform a sensitivity analysis by simultaneously varying some relevant indicators.

In the environmental dimension, the indicators are based on the mass and chemical properties of the substances involved. According to approach adopted in this study, they are not considered sensitive, as these parameters are intrinsic and cannot be modified. In contrast, the economic dimension is influenced by market fluctuations. Therefore, the “Cost” and “Revenue” indicators were varied by adding the price differences (USD/kg) between the 10th and 90th percentiles (P10 and P90), calculated from historical data between September 2024 to September 2025.^43,44^ In the social dimension, only the “RT” and “RP” indicators were modified, using the minimum and maximum reported values of operating temperature and pressure.^16,39–41,45^ Finally, the technological dimension was not varied, as the highest reported TRL value was assumed.

Two scenarios were generated to evaluate these fluctuations. The Negative Variation scenario models a decrease in the overall index representing a more sustainable outcome, this is due to a reduction in the “Cost”, “RT”, and “RP” indicators alongside an increase in “Revenue”. Conversely, the Positive Variation scenario reflects an increase in the index, leading to a less sustainable outcome driven by higher “Cost”, “RT”, and “RP” values combined with a minimum “Revenue” value. This approach keeps the original logic of the sustainability variation for the selected indicators. The results are presented in Figure 2.

The results of this analysis indicate that sustainability values remain largely unchanged, preserving the ranking of alternatives observed in the baseline scenario. Sustainability index is only minimally affected by price fluctuations, which is consistent with the relative stability of B2B chemical markets, where prices are primarily influenced by major global geopolitical events. In contrast, variations in operating conditions such as temperature and pressure exhibit more noticeable deviation; however, the resulting index changes remain small because these conditions fluctuate within relatively narrow ranges (on the order of 10^2^–10^3^), with no extreme operating conditions reported.

To better understand these results, the relative influence of each dimension on the overall sustainability index was evaluated for the base case. This influence was determined by considering the individual contributions of each dimension prior to aggregation and calculating the percentage of contribution to the total index. This approach allows the identification of the dimension with the greatest impact on the sustainability index for each alternative. The resulting distribution is presented in Figure 3.

**Figure 3:**
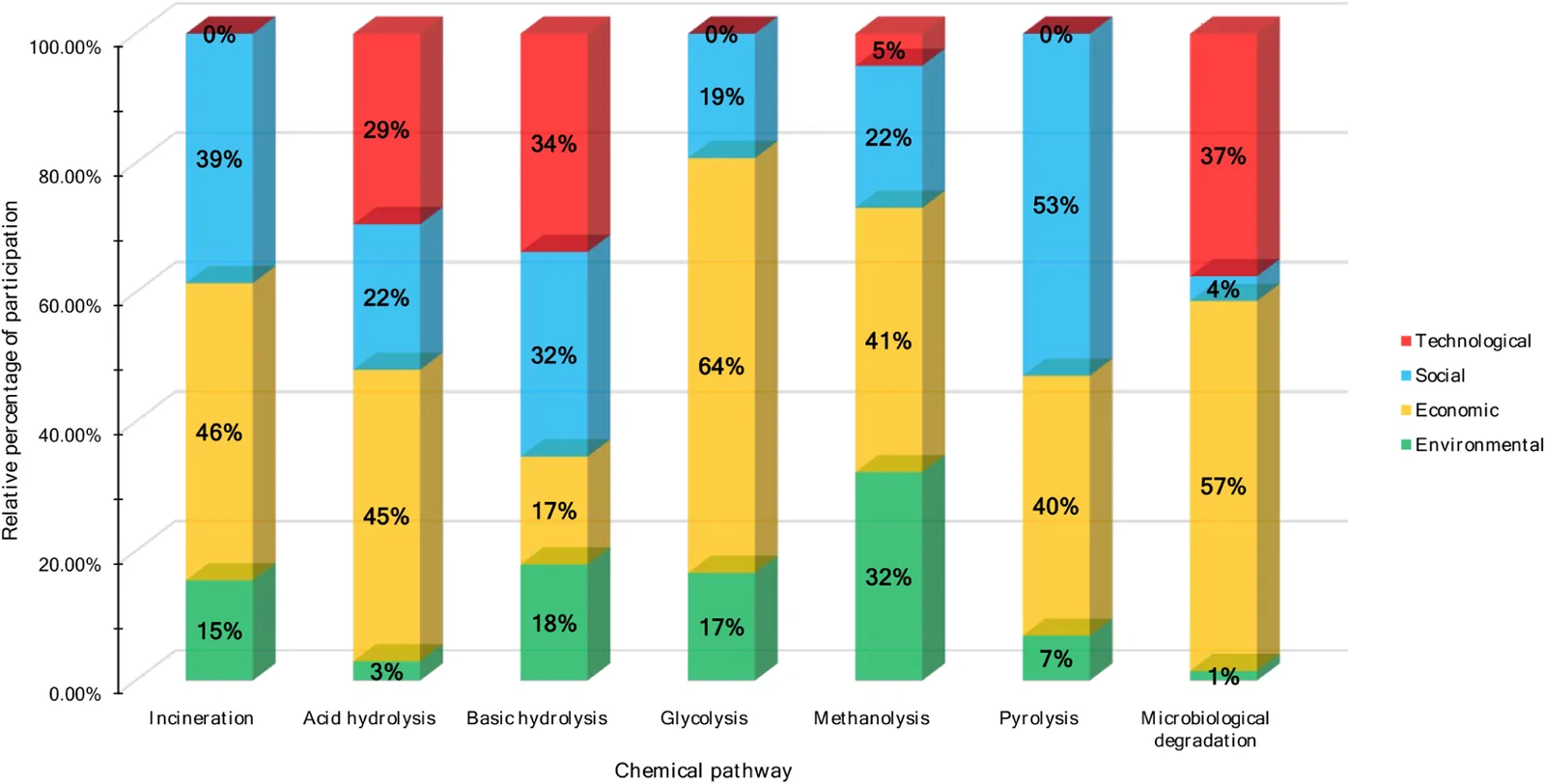
Contribution of each dimension to the sustainability index under the base case for PET chemical valorization sustainability assessment.

As shown in Figure 3, the economic dimension highlights as one of the strongest influences on the index. This is primarily supported by the normalized value of this dimension reported in Table 5, where the distribution of the economic indicators, particularly “Revenue”, is notably unfavorable for all alternatives except methanolysis. From a sustainability perspective, this indicates that economic factors represent the primary source of impact across the evaluated pathways. Consistent with the analysis, glycolysis and microbiological degradation are the pathways most strongly influenced by the economic dimension with contributions of 64% and 57%, respectively; these values are higher but close to the prevalent alternative incineration (46%). As the economic dimension considers the purchase and sale of the substances involved in the reaction, efforts toward the implementation of these most sustainable alternatives should focus on reducing the raw materials costs. Therefore, these pathways will become more sustainable by making technology more affordable, thus, the implementation of strategies aimed at reducing raw materials consumption by increasing conversion and yield through the selection of adequate reaction conditions, more active and selective catalysts or by implementing process intensification concepts should be explored for making glycolysis and microbiological degradation more competitive from the economic dimension.

The environmental dimension shows the lowest influence on the index, except for the methanolysis alternative. This value is atypically high due to the large amounts of dichloromethane and methanol required in this pathway, both of which pose significant environmental, toxicity, and flammability risks. This characteristic has been identified as a limitation of that alternative, as achieving high yield (>98%) requires large amounts of methanol and dichloromethane relative to the quantity of PET processed.^40,46^ Although several studies have explored this pathway attributable due to its short PET processing time and the potential for direct integration into the current PET industrial production processes, it is often considered an obsolete technology. This is because of the complex challenges in separation stages which make nonpractical its implementation.^22^ To mitigate solvent consumption, catalysts such as potassium carbonate (K_2_CO_3_) and nanometric zinc oxide dispersions have been explored, along with alternative approaches including microwave-assisted and supercritical fluid techniques.^16,47^ However, these strategies may lead to increased reaction times and do not necessarily increase the sustainability of the process. Therefore, further efforts should focus on exploring the use of green solvents that can replace dichloromethane and mitigate its negative impact. Lastly, microbial degradation, despite being one of the three highest-ranked alternatives, is also the least developed as reflected in the contribution of the technological dimension, which represents a significant disadvantage for its implementation.

### Sensitivity Analysis of Weight Distribution

A recognized opportunity for improving sustainability assessment lies in the involvement of stakeholders, who often assign varying levels of importance to criteria based on their respective roles and perspectives.^48^ To deal with this gap and to evaluate how the weights influence the outcomes, a sensitivity analysis was performed extending the base case to account for variations in the relative weighting of each dimension. Thus, the sustainability index values were evaluated over an established weighting range of 20–75% for each dimension as shown in Figures 4–7. According to these results, methanolysis consistently remains the least sustainable option regardless of the weighting scheme applied, barely managing to improve one position in cases where relative weights become extreme (∼60–70%). Sensitivity analyses prioritizing social or environmental dimensions show minimal variation in the ranking of the most sustainable alternative, with glycolysis and microbiological degradation consistently identified as the best ranked options.

**Figure 4:**
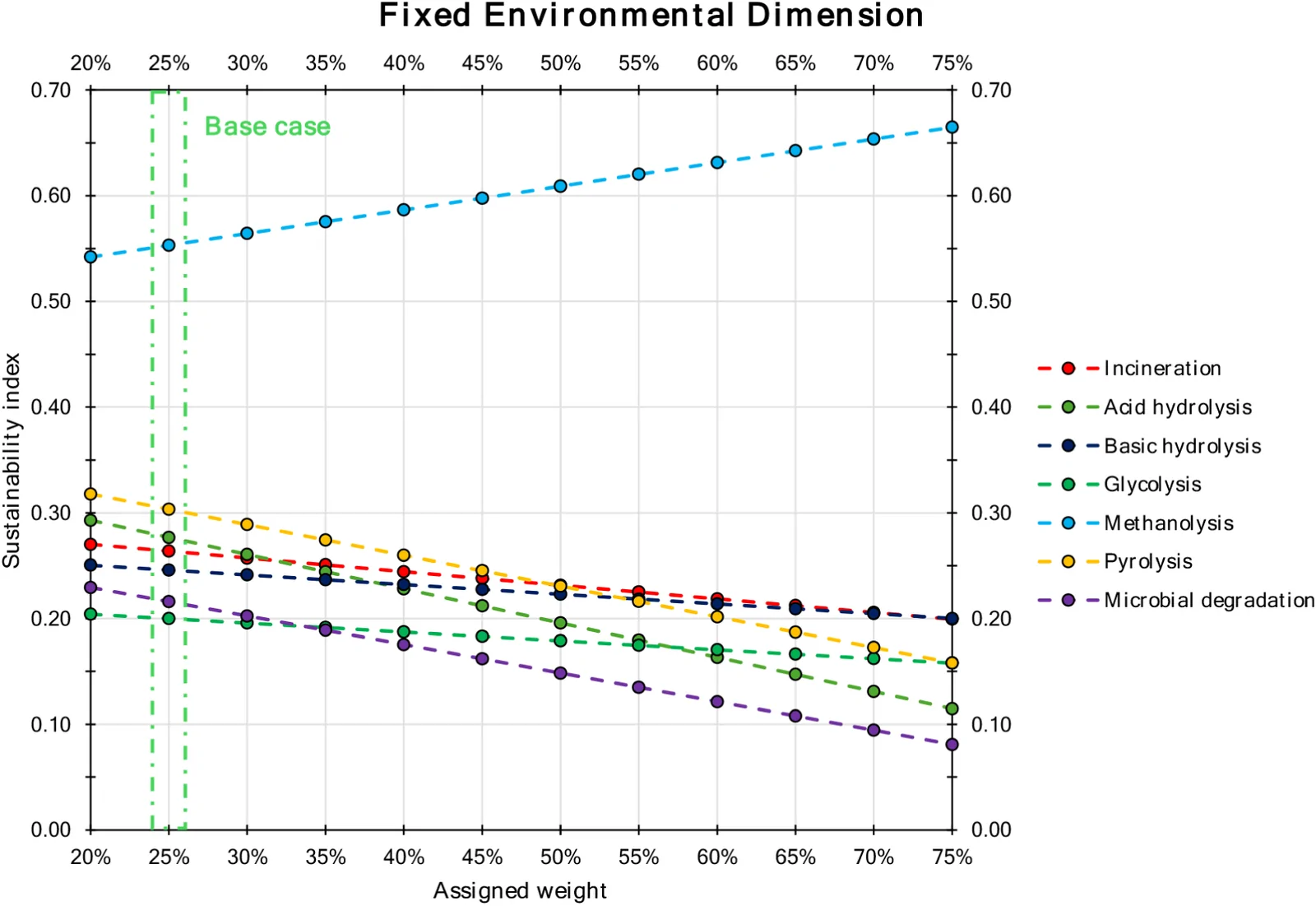
Comparison of the sustainability index behavior as a function of the environmental dimension.

**Figure 5:**
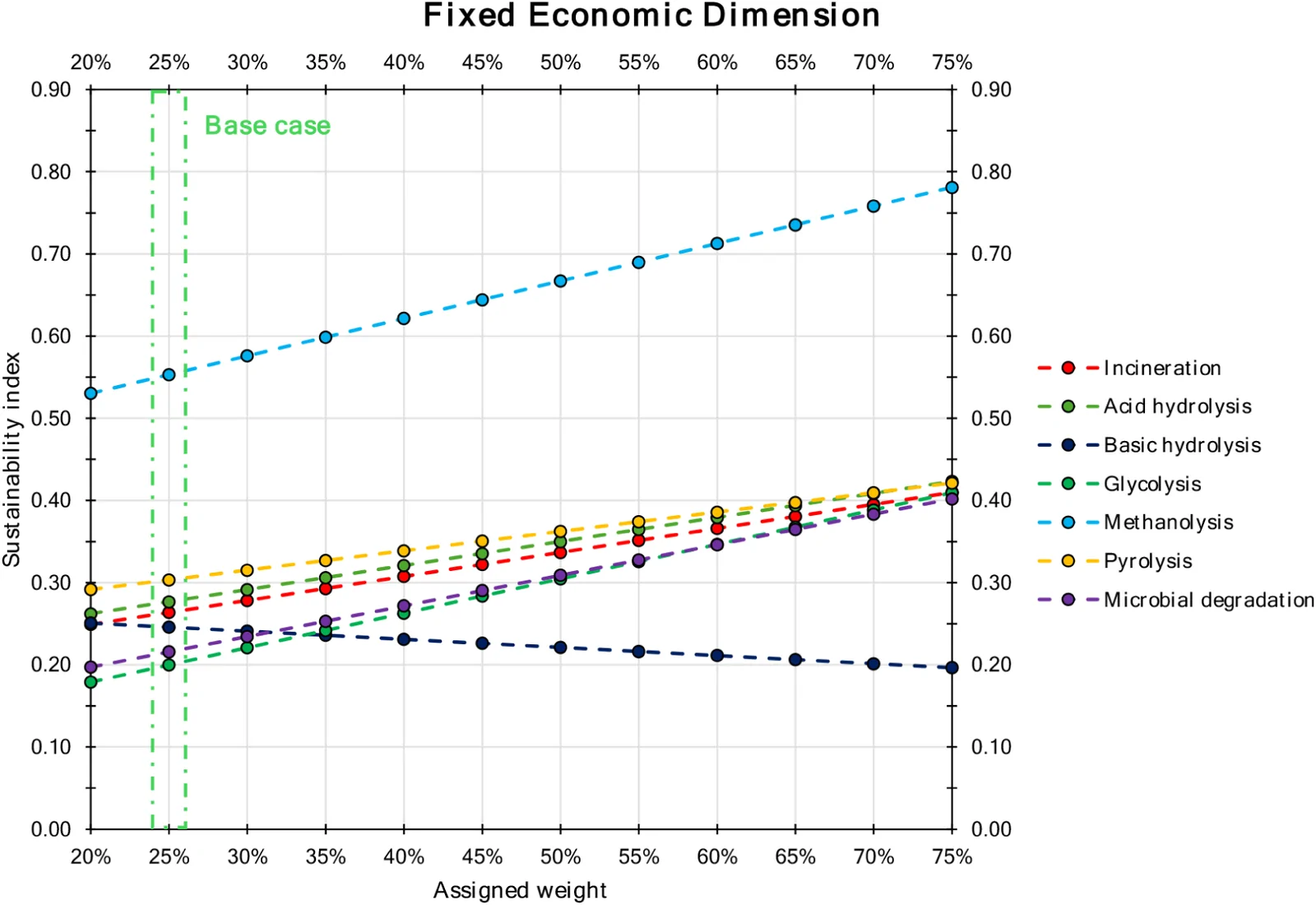
Comparison of the sustainability index behavior as a function of the economic dimension.

**Figure 6:**
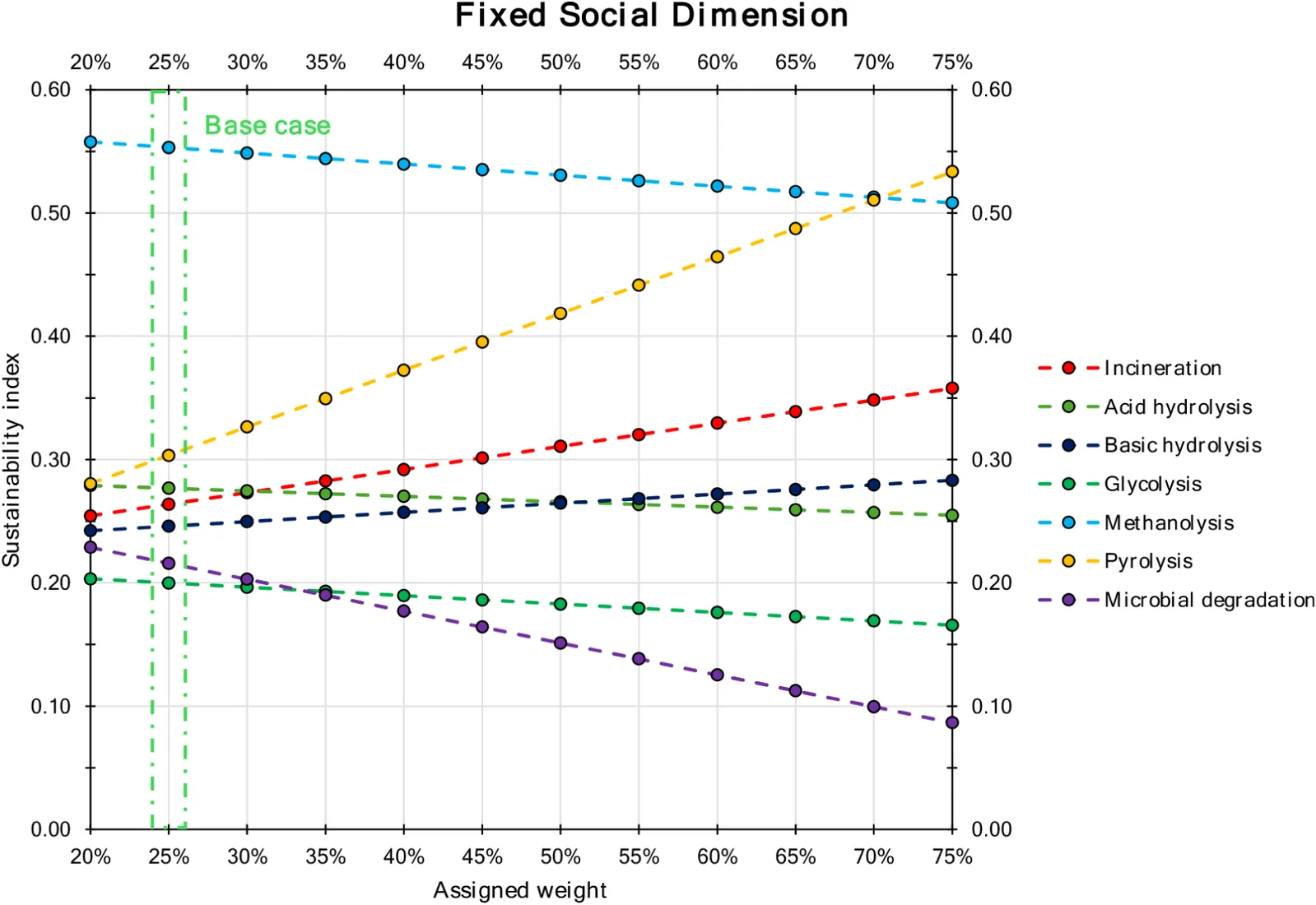
Comparison of the sustainability index behavior as a function of the social dimension.

**Figure 7:**
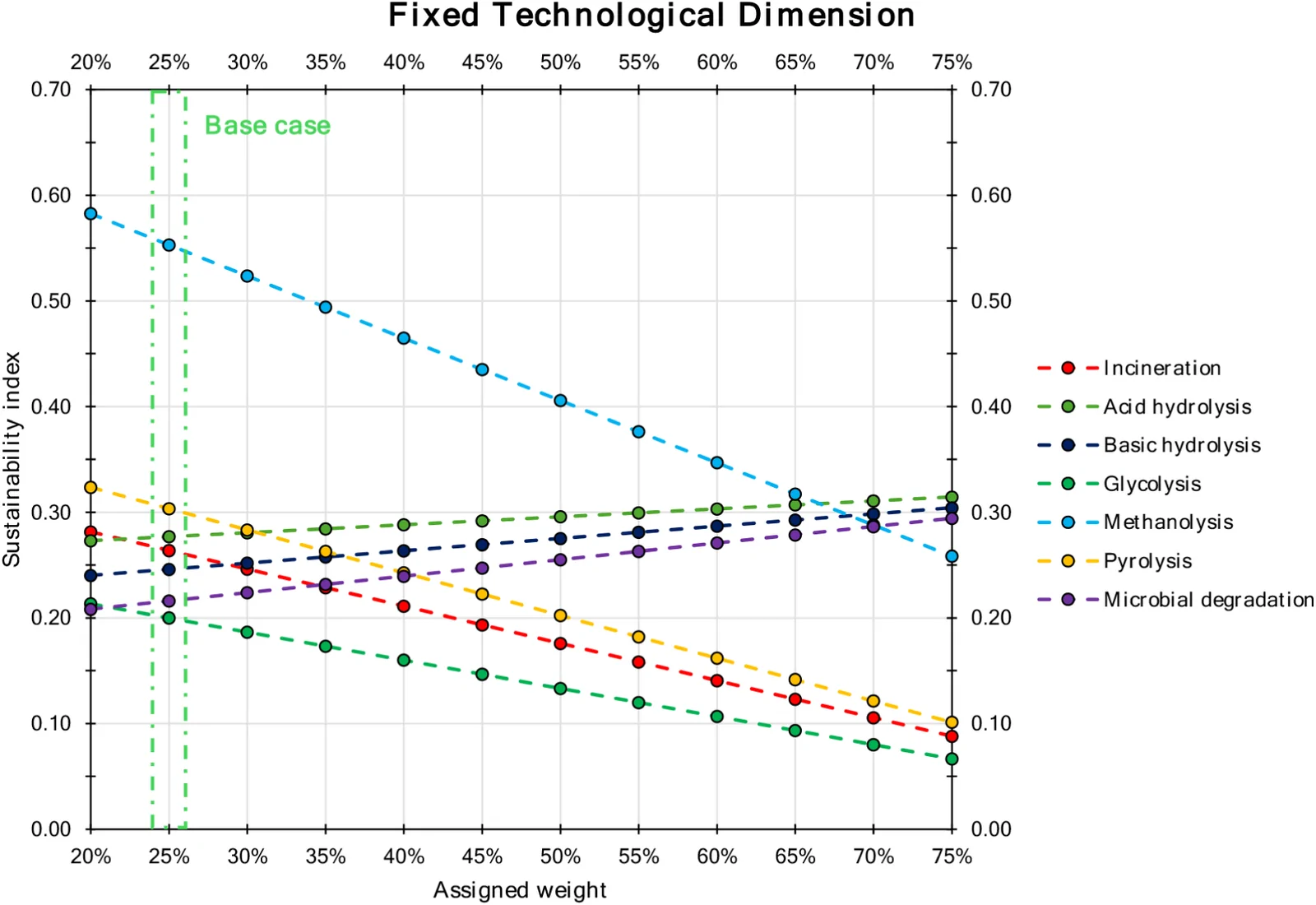
Comparison of the sustainability index behavior as a function of the technological dimension.

Figure 4 presents scenarios where the weight assigned for environmental dimension is dominant. It is observed that when its contribution exceeds 35%, microbial degradation pathway (0.10 < SI < 0.20), followed closely by glycolysis, is favored because of the restrictions on hazardous chemicals. Acid hydrolysis surpasses basic hydrolysis in weightings close to 40% (∼40%; SI ∼ 0.20) for then surpassing glycolysis at the upper end of the weighting range (<60%). This trend can be explained by the fact that, as environmental importance increases, the involvement of a larger number of chemical substances increasingly hinders the positive evolution of sustainability.

Both hydrolysis processes (acid and basic) remain favorable, below incineration, in low-to-medium environmental importance scenarios (20% < $w_{i}^{k}$ < 50%). However, basic hydrolysis tends to have an impact identical to that of incineration in scenarios of high environmental importance, due to its high chemical consumption (SI ∼ 0.20). A similar pattern is observed for glycolysis and pyrolysis, which converge to comparable values in the most extreme scenario (<0.15). This behavior suggests that, from a chemical perspective, use of mixtures of different substances may ultimately result in a similar environmental impact.

Overall, as shown in Figure 5, all pathways became less favorable as the importance of the economic impact increases, except for basic hydrolysis, which consistently benefits from a higher economic weighting decreasing the index value from ∼0.35 to ∼0.20. This is likely due to increased revenue from the sale of its byproduct, sodium sulfate. This byproduct would inevitably be produced in large quantities because of the reaction stoichiometry and can lead to additional profits considering its stable and growing market in recent years (CAGR ∼4.5%).^43,44^

Despite this general trend, the three most sustainable alternatives identified in the base case remained consistently as the most favorable. In general, the pathways exhibit increased unsuitability, with an increase of the SI of approximately 0.20 points, except for methanolysis, which shows a larger worsening of about 0.30 points. This seems to suggest that, in a practical implementation perspective, most pathways (except for methanolysis) have broadly comparable economic impacts (SI ∼ 0.40). Consequently, the main challenge in implementation and design lies in optimizing the separation stages to maximize the obtention of products once an alternative has been selected.

From a social perspective, as shown in Figure 6, the routes identified as favorable from the environmental point of view maintain the trend at social weight values of 30% or higher. The two hydrolysis routes display similar levels of favorability within a common range (∼0.25 < SI < 0.30) across variations in social weighting. This is expected, since both routes involve similar substances (e.g., sulfuric acid, ethylene glycol, and terephthalic acid), although the acid route is slightly favored due to the lower mass contribution of substances involved. In contrast, incineration and pyrolysis exhibit the most pronounced increase becoming less favorable (∼0.35 < SI < 0.40 and ∼0.35 < SI < 0.55, respectively), which is consistent with expectations, given that the reaction products generated in these processes are highly harmful to both the environment and human health, affecting not only workers but also the broader population. While these methods may appear advantageous from a traditional technical-economic perspective, they could generate conflicts in future implementation if only that dimension is considered. This highlights the importance of incorporating the social aspect from the early stages of design.

Finally, Figure 7 illustrates the influence of the technological dimension, which, despite being defined only by the TRL, represents the most relevant aspect for scaling up to a feasible process. The observed trend is straightforward, recycling technologies with higher TRL values achieve better sustainability ratings as the weight of the technological dimension increases. As the relative weight assigned to this dimension rises, two distinct groups emerge: incineration, glycolysis, and pyrolysis, with SI values of approximately 0.10, and acid and base hydrolysis, along with microbial degradation, with SI values of approximately 0.30. Methanolysis stands out, reaching a final SI of approximately 0.25, representing the only scenario where outperforms other alternatives.

### Selection of the Most Sustainable Pathway

As shown in Figures 3–**7**, methanolysis consistently stands out as the least sustainable pathway among the seven evaluated. The next less sustainable option is pyrolysis, which also appears to perform worse than incineration. This aspect is particularly significant, because it highlights that not all chemical alternatives can be foreseen as a feasible replacement for PET waste incineration from a sustainability perspective. Under this premise, if incineration is to be regarded as a transitional alternative, it is crucial to position a range of chemical recycling processes as a potential near-term solution, while systematically evaluating each alternative based on sustainability criteria to ensure a sustainable transition.

After the analysis, glycolysis consistently results in the most sustainable pathway for recycling PET plastic waste, continuously remaining above incineration, except in the case where it is the technological dimension that has the greatest level of importance. This is expected since all tertiary alternatives have reported TRL values lower than general waste incineration. Figure 8 summarizes the overall behavior across dimensions of sustainability index value for the three most sustainable pathways and PET waste incineration.

**Figure 8:**
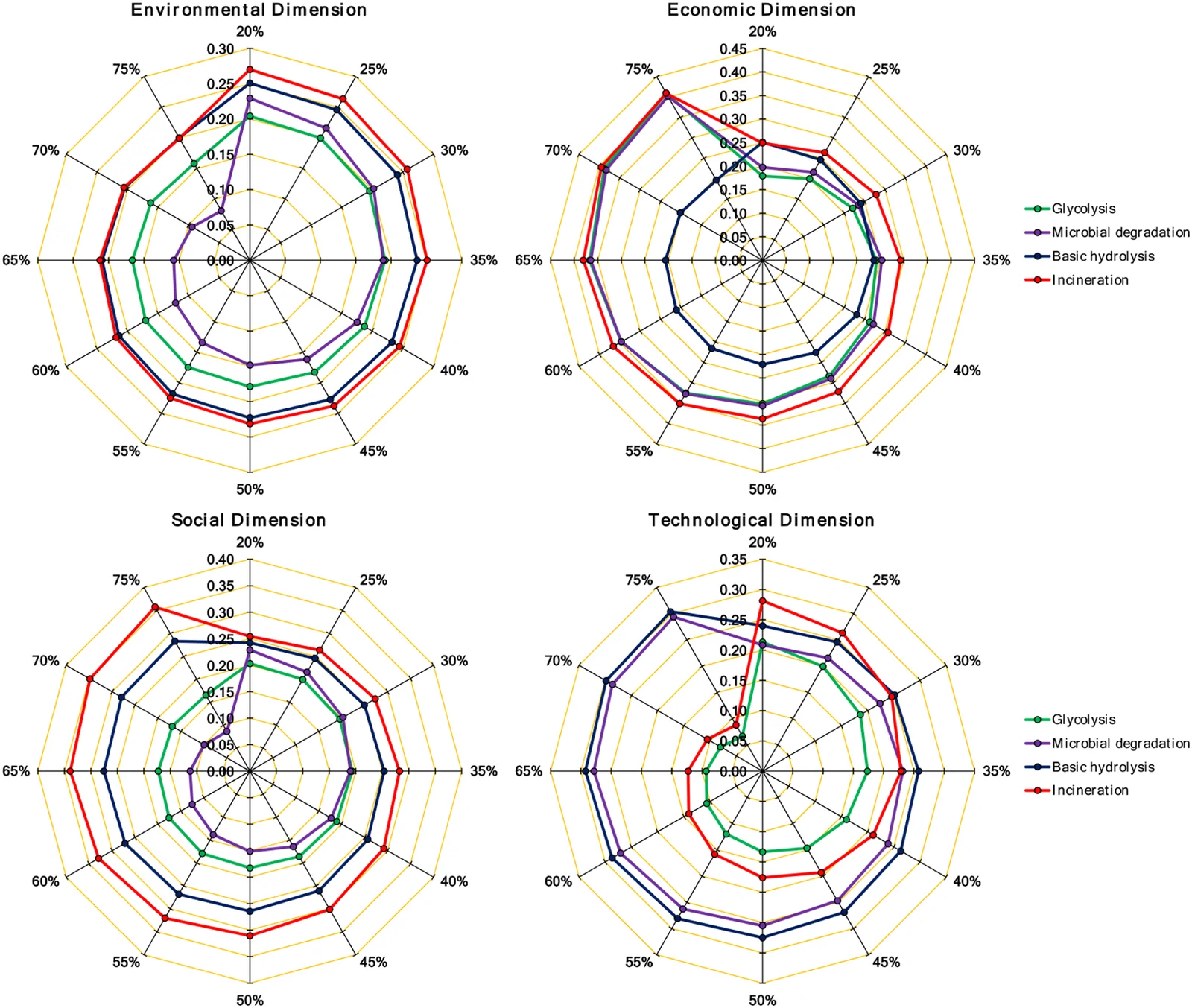
Comparison of the sustainability index behavior across dimensions for the three most sustainable routes against PET waste incineration.

Some studies support the use of glycolysis for processing PET waste. For example, Lang et al. conducted a comprehensive review of chemical recycling methods for PET waste using chemical pathways, analyzing processes under various operating conditions such as temperature, pressure, catalysts, and reactant ratios, as well as the use of advanced technologies like microwaves and ultrasound.^49^ Their comparative approach was based on green chemistry metrics, concluding that glycolysis is the most balanced method in terms of efficiency and under moderate conditions.^49^ Babaei et al. described glycolysis as a technology that has been updated over time by adding eco-friendly catalysts and addressing the challenges of reaction times and low yields, hindering its implementation at an industrial scale.^47^ Uekert et al. presented a comprehensive quantitative framework to evaluate plastic recycling technologies by integrating technical, economic, and environmental indicators into a multicriteria decision analysis. They concluded that glycolysis represents a viable chemical alternative that offers favorable performance for process implementation.^50^

Glycolysis, from processing point of view, is a promising PET waste treatment and recycling process due to its reliability and simplicity to degrade this polymer.^51^ Although this study selected ethylene glycol as the reaction medium, other types of glycols such as diethylene glycol or propylene glycol^47^ can be used to start it up, being one of the many adaptive features that make this process attractive for its application. For example, this process usually operates at relatively low temperatures (180 °C–240 °C) and obtains high polymer conversion rates.^16^ Since it is a slow-occurring process, it is typically catalyzed by a wide variety of substances such as organometallic compounds of zinc, cobalt, and manganese, in addition to zeolitic beds. Other alternatives have also been investigated such as catalysis using nano catalysts made of metallic oxides of carbon, iron, zinc, or cobalt,^52^ start-up in supercritical conditions assisted by microwaves^16^ or realization in other reaction media such as ionic liquids or eutectic solvents.^47^ This demonstrates that the wide range of research developed around this alternative aligns with the fact that PET glycolysis is currently the most technologically studied alternative, typically focused on tackling the waste that could not be treated by mechanical recycling.^53^ This places this alternative as a backup to secondary recycling but, in the near future, it could eventually replace it, thus converting mechanical recycling into either a transitional alternative or at least into an alternative that complements chemical processes. Figure 9 represents graphically the PET waste valorization following the incineration and glycolysis routes.

**Figure 9:**
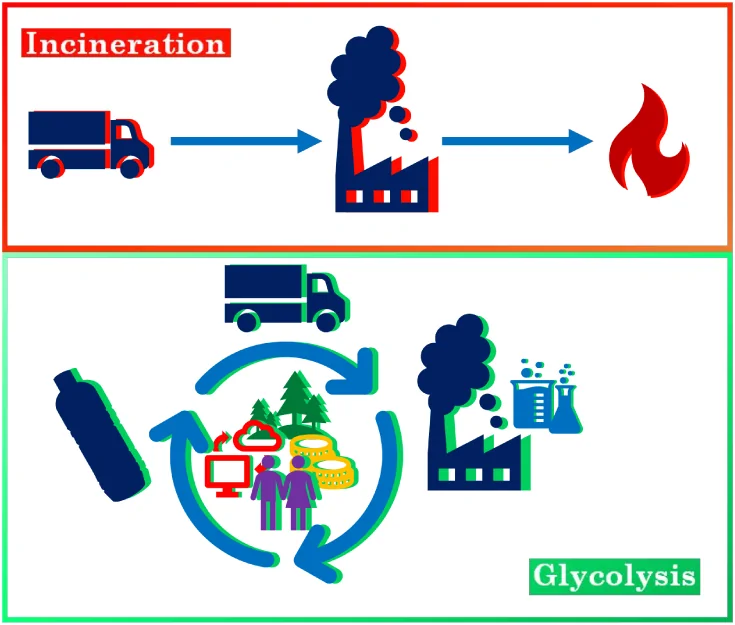
Graphic representation of PET wastes valorization by incineration and glycolysis.

Another promising alternative is basic hydrolysis, which turns out to be one of the least impacted on the weight variation analysis. This alternative is more attractive in the economic dimension than glycolysis. However, this result is related to the inclusion of sodium sulfate (Na_2_SO_4_) as a recoverable byproduct of the neutralization of terephthalic acid salt. Considering the separation and purification costs of this byproduct may result in a modification of the impact of the added value (Revenue) of this alternative. Therefore, this aspect emerges as a relevant for future analysis.

Although microbiological degradation appeared to be one of the most sustainable alternatives throughout the designed evaluation, relevant factors such as prereaction equipment conditioning, or reaction time (14 days for the bioprocess with the selected fungal species) were not considered. On an industrial scale, this process presents a disadvantage due to its batch or semicontinuous nature, as it requires a long residence time in the reactor, limiting the amount of plastic processed compared to a continuous process with a constant flow of plastic. Furthermore, the sensitivity of the microorganism to environmental and operational conditions, the size of the reactors required, the additional nutrients required, and the proper disposition of active microorganisms in the natural environment are factors that can affect the interpretation of the results. In addition, it should be noted that the sustainability assessment proposed considers aspects related to the chemical reaction processes, particularly the substances involved, that is why microbiological degradation is slightly affected in the environmental and social dimensions. However, the consideration of this pathway is due to the recognition as an active field of research, innovation, and development with respect to the other tertiary alternatives, so it is valid to include it among the current options for the problem of plastic waste, in this case PET residue.

### Contributions and Limitations of the Assessment Framework and Recommendations for Scale-Up

Compared to mechanical recycling, either in situ re-extrusion or pelletizing, the resulting product is a polymer with lower mechanical quality than the virgin resin, whereas chemical treatment allows the company that implements it to produce of a wide variety of products because the monomers can be obtained and reentered into the value chain to remanufacture the original polymer or to be used as raw material in other industries.^16,47^ Chemical recycling processes could prevent the release of contaminants directly into the earth or groundwater reserves with respect to the sanitary landfill.^16^ Therefore, the range of use of the waste and valorization of the original product is higher, promoting a circular approach and then a more sustainable environment for these recycling processes. This is not just an appreciation, since it is known that tertiary recycling processes for PET reduce a considerable amount of greenhouse gas emissions compared to virgin PET production by providing a carbon-neutral alternative over primary and secondary pathways due to the processing of contaminated waste (unsuitable for mechanical processing).^47^ By extension, tertiary category represents more sustainable alternatives than the quaternary category where its main disadvantage is precisely the release of combustion gases into the atmosphere.^16^ Consequently, this study is valuable because it evaluates and compares these types of alternatives simultaneously from a sustainability point of view integrating technical, economic, and environmental indicators.

The proposed assessment provides an approach to guide an informed decision-making process by evaluating both advantages and disadvantages through an index that enables the identification of key factors influencing sustainability performance. The results highlight the technological benefits of chemical recycling over primary, secondary, and quaternary alternatives, including its advantages compared to incineration. With the proposal of a multidimensional sustainability assessment of major recycling pathways, it was possible to identify their critical impacts in specific scenarios leading to recognizing improvement opportunities. At the same time, this study reveals key challenges, such as the risks inherent to chemical processes, the need to adopt greener solvents and catalysts, and the gap between technological development and large-scale industrial applications. These aspects should be then prioritized to improve the sustainability of plastic recycling systems thereby supporting the mitigation of potential negative impacts from early design to full-scale implementation.

As the evaluation was conducted gathering data provided by different studies; with the consideration of characteristics (reagents, solvents, catalysts) most common reported in literature for each alternative, the results are tied to the information collected. The proposed index lead to the evaluation of the technological characteristics of the recycling pathways themselves, whereas feedstock heterogeneity and pretreatment requirements are system-dependent aspects that should be incorporated during subsequent process design and case-specific assessments. Consequently, it is necessary to remember that this type of study should be considered as a guide or initial approach to support decisions in the planning stages for future related projects in this area.

This study establishes a first sustainability assessment for the selection or consideration of chemical recycling processes, focusing entirely on their characteristic reaction. Therefore, the next step is to expand the evaluation scenario to include more information in a process-oriented view, for example, adding aspects such as kinetics and thermodynamics as well as the subsequent separation and purification stages, specially focusing on the most promising alternatives identified in this study. Likewise, it would be interesting to assess the most sustainable pathways from this study on a larger scale either experimentally or under evaluation in simulated environments, considering some variants of each pathway described, to progress toward a solution industrially applicable and effective. To achieve that goal, further techno-economic studies are recommended for the implementation of PET recycling technologies based on hybrid approaches combining mechanical and chemical solutions, as a pathway toward a consistent industrial transition to more sustainable applications.

## Conclusions

In this study, seven PET waste recycling pathways were evaluated under a sustainability assessment designed to consider elements inherent to environmental risks, economic valorization, social safety in the handling of chemicals, and the technological state of development, thus addressing environmental, economic, social, and technological dimensions. The contribution of this study is focused on the application of a sustainability assessment in chemical transformation pathways from an early design stage perspective due to their potential compared with classic mechanical alternatives such as pelletization or extrusion. In this evaluation, a baseline scenario was established with a similar level of importance for all considered dimensions. Sensitivity analysis was conducted with a varying distribution within a defined range. As a result, glycolysis was found to be the most sustainable alternative, standing out in most of the proposed scenarios as the lowest impact pathway. Moreover, basic hydrolysis and microbiological degradation are other promising proposals that would address the use of incineration as an existing solution to the PET waste disposal problem. The analysis is based on secondary data from the literature and explicitly acknowledges uncertainties related to data sources and pathway definition. Overall, the results indicate that it is possible to transition from the classic approach of mechanical recycling or burning of waste, to one of chemical reintegration into the value chain, with a wide range of possible pathways for its development, updating, and implementation. The results support the need to update chemical recycling technologies to reach a point where their application on an industrial scale is feasible, while maintaining a vision that takes care of every dimension to ensure sustainable life cycle processes. Further studies can build upon the results obtained to gain a more precise understanding of pathways and guide more informed decision-making for PET recycling.

## Supplementary Material



## Notes

Data about technologies and indicators found in the literature review is compiled as Supporting Information. Similarly, all information collected and used for the mass balance calculations as well as the technical data related to the operating conditions of the recycling pathways, properties related to chemical hazards of the substances, and economic data on market purchase and selling prices are also available on this material (PDF)
