# Supplementary material for: Multidimensional Sustainability Assessment of Chemical Valorization Pathways for PET Waste: A Literature-Based Early-Design-Stage Index Including Technology Readiness
Source: ACS Omega. 2026 Aug 14;11(33):49280–98. doi: 10.1021/acsomega.6c02141 (PMC13519995; doi:10.1021/acsomega.6c02141)
Supplement: Supplementary file 1 [file ao6c02141_si_001.pdf]

Multi-dimensional Sustainability Assessment of Chemical Valorization Pathways for PET Waste: A Literature-Based Early-Design-Stage Index Including Technology Readiness

Daniel Alberto Pulido-Peña †, Luis López Rojas †, Paulo César Narváez-Rincón †,*

† Grupo de Procesos Químicos y Bioquímicos, Departamento de Ingeniería Química y Ambiental, Universidad Nacional de Colombia, Carrera 30 45-03 - Edificio 412, Bogotá, 111321, Colombia.

* Corresponding author.
E-mail address: pcnarvaezr@unal.edu.co (Paulo César Narváez-Rincón).
Phone number: +57 6013165000 Ext. 14301

Supplementary Material

Table S1. Technologies for PET valorization and their classification according to their recycling category. 2

Table S2. Initial list of the forty-eight (48) sustainability indicators........ 3

Table S3. Net mass balance for 1 ton of pure PET calculated using the PET/substance ratio involved in the selected alternatives for chemical valorization of PET. 4

Table S4. Reported temperature and pressure conditions for the selected alternatives for chemical valorization of PET. 4

Table S5. H statements and NFPA 704 values of the substances involved in the selected alternatives for chemical valorization of PET. 5

Table S6. Price per kilogram of the substances involved in the selected alternatives for chemical valorization of PET, according to United States market. 6

Table S7. Technological Readiness Level (TRL) of the selected alternatives for chemical valorization of PET. 6

Table S8. Indicators matrix from raw data used for sustainability assessment. 7

Figure S1. Comparative figures by indicator of the proposed normalization methods. 8

References 16

Table S1. Technologies for PET valorization and their classification according to their recycling category.

Technology	Category	Definition	Reference
Open fill	-	Disposal of plastic waste by storing it on land without prior treatment of the waste.	[1]
Sanitary fill	-	Disposal of plastic waste by sequentially storing it between layers of waste covered by layers of soil that are compacted within a previously prepared space.	[1], [2]
Incineration	Quaternary	Combustion of plastic waste, using it as a source of fuel for the obtention of heat or as a means of thermal energy generation via water vaporization. Usually, it represents the last and most convenient alternative of waste disposal when the other means cannot be afforded	[1], [3], [4]
Pelletizing	Secondary	Processing the treated and cleaned plastic waste by melting for its conversion by extrusion into raw material for lower category plastic use, because this process alters the mechanical qualities of the polymer, making it less resistant.	[1], [4]
Hydrolysis	Tertiary	Slow depolymerization of the clean PET residue via reaction with water molecules in conditions of high pressures and temperatures. It can be done in acidic, neutral, or basic mediums for the obtention of terephthalic acid and ethylene glycol, for their posterior conversion into raw materials for other purposes.	[4], [5]
Glycolysis	Tertiary	Fast and low cost depolymerization of the clean PET residue, reacting with ethylene glycol to produce bis(2-Hydroxyethyl) terephthalate (BHET) and other terephthalic glycols that can be used in the manufacturing of diverse polymers such as unsaturated resins, polyurethane foams, or acrylic covers. The reaction can be done with the use of a catalyst, or a solvent, in supercritical conditions or assisted by microwave radiation.	[4], [5]
Methanolysis	Tertiary	Use of methanol for the conversion of PET residue to dimethyl phthalate releasing ethylene glycol. The reaction is conducted under high pressures and temperatures, obtaining two monomers useful for the repolymerization of new PET. It is usually catalyzed with organometallic substances, especially acetate types.	[4], [5]
Pyrolysis	Tertiary	Decomposition of plastic waste via the direct heat at elevated temperatures in an inert atmosphere, using heating assisted by plasma or microwave radiation. It produces different organic products such as fuel oils or valuable combustion gases.	[1], [6]
Open loop	Secondary	Recycling PET waste through mechanical and chemical processes for direct production or through the prior manufacture of pellets, to obtain plastic fibers that will be used in the manufacture of new lower category plastic products.	[1]
Close loop	Secondary	Recycling uncontaminated PET waste through mechanical processing to obtain plastic material with characteristics similar to the original plastic, in order to manufacture the product from which it was originally discarded.	[1]
Microbiological degradation	-	Chemical decomposition of the polymer assisted with bacterial or fungi species taking advantage of the enzymatic activity of the microorganism, which can be found in nature or be genetical modified (GMO).	[7]

Table S2. Initial list of the forty-eight (48) sustainability indicators.

Dimension	Indicators	Reference
Environmental	Abiotic Depletion Potential	[3]
	Air pollution	[1]
	Biodiversity	[1]
	Climate change	[1]
	Eutrophication Potential	[3]
	Freshwater Ecotoxicity Potential	[3]
	Global Warming Potential	[3]
	Land use	[1]
	Marine Ecotoxicity Potential	[3]
	Ozone Depletion Potential	[3]
	Photochemical Oxidant Formation Potential	[3]
	Soil pollution	[1]
	Terrestrial Acidification Potential	[3]
	Terrestrial Ecotoxicity Potential	[3]
	Water pollution	[1]
Economical	Cost	[3]
	Gate fee	[1]
	Initial investment cost	[1]
	Operation and management cost	[1]
	Revenue	[3]
Political	Administrative incentive	[1]
	Public organization	[1]
	Regulation	[1]
Social	Child labor	[8]
	Community engagement	[8]
	Contribution to Economic Development	[8]
	Discrimination	[8]
	Fair salary	[8]
	Forced labor	[8]
	Health and safety	[8]
	Human health	[1]
	Human Toxicity Potential	[3]
	Job creation	[1]
	Public acceptance	[1]
	Public participation	[1]
	Social Benefit	[8]
	Waste prevention behavior	[1]
Technological	Avoided chemical production	[1]
	Capacity	[1]
	Energy consumption	[1]
	Feasibility	[1]
	Maturity	[1]
	Product value	[1]

Table S3. Net mass balance for 1 ton of pure PET calculated using the PET/substance ratio involved in the selected alternatives for chemical valorization of PET.

Route	Substance	Mass involved (t)	Data reference*
Incineration	Carbon dioxide	1.4	[9]
	Ammonia	2.7×10^{-5}	
	Lead	8.4×10^{-8}	
	Cadmium	7.5×10^{-9}	
	Mercury	1.0×10^{-8}	
	**Total**	**1.4**	
Acid hydrolysis	Terephthalic acid	0.22	[10]
	Ethylene glycol	0.08	
	Sulfuric acid	2.04	
	**Total**	**2.35**	
Basic hydrolysis	Terephthalic acid	0.22	[11]
	Ethylene glycol	0.08	
	Sodium hydroxide	12.0	
	Sulfuric acid	14.7	
	Sodium sulphate	21.3	
	**Total**	**48.3**	
Glycolysis	Ethylene glycol	1.11	[4], [12]
	Bis(2-hydroxyethyl) terephthalate	0.34	
	Zinc acetate	0.01	
	**Total**	**1.47**	
Methanolysis	Methanol	7.70	[12]
	Dimethyl terephthalate	1.01	
	Ethylene glycol	0.32	
	Zinc acetate	0.01	
	Dichloromethane	7.69	
	**Total**	**16.7**	
Pyrolysis	4-(vinyloxycarbonyl) benzoic acid	0.27	[13]
	Acetaldehyde	0.21	
	Benzoic acid	0.10	
	Ethylene glycol dibenzoate	0.05	
	Vinyl benzoate	0.05	
	Divinyl terephthalate	0.05	
	Ethynylbenzene	0.04	
	Terephthalic acid	0.03	
	Benzene	0.03	
	**Total**	**0.82**	
Microbiological degradation	Terephthalic acid	**0.28**	[14]

*Source of extracted data.

Table S4. Reported temperature and pressure conditions for the selected alternatives for chemical valorization of PET.

Alternative	Temperature (°C)	Pressure (bar)	Reference
Incineration	500 - 1000	1	[15]
Acid hydrolysis	180	14 - 24	[4], [16]
Basic hydrolysis	210 - 250	14 - 24	[4]
Glycolysis	180 - 240	1	[4]
Methanolysis	180 - 280	20 - 40	[4]
Pyrolysis	300 - 600	1	[13]
Microbiological degradation	60	1	[14]

Table S5. H statements and NFPA 704 values of the substances involved in the selected alternatives for chemical valorization of PET.

Alternative	Substance	H statements	NFPA:F*	NFPA:R**
Incineration	Carbon dioxide	280 281	0	0
	Ammonia	221 280 314 331 332 335 400 411	1	0
	Lead	302 332 360 362 372 373 400 410	-	-
	Cadmium	250 302 330 335 341 350 361 372 400 410	-	-
	Mercury	290 300 330 360 372 400 410	-	-
Acid hydrolysis	Terephthalic acid	315 319 335	1	0
	Ethylene glycol	302 373	1	0
	Sulfuric acid	290 314	0	2
Basic hydrolysis	Terephthalic acid	315 319 335	1	0
	Ethylene glycol	302 373	1	0
	Sodium hydroxide	290 314 315 318 319	0	1
	Sulfuric acid	290 314	0	2
Glycolysis	Ethylene glycol	302 373	1	0
	Zinc acetate	302 318 319 400 410 411	1	1
Methanolysis	Methanol	225 301 311 331 370	3	0
	Dimethyl terephthalate	317 319 412	1	0
	Ethylene glycol	302 373	1	0
	Zinc acetate	302 318 319 400 410 411	1	1
	Dichloromethane	302 315 319 335 336 341 351 373	1	0
Pyrolysis	Acetaldehyde	224 302 311 317 319 335 341 350 351	4	2
	Benzoic acid	302 315 318 319 335 372	1	0
	Ethylene glycol dibenzoate	411	-	-
	Vinyl benzoate	315 317 319 335 410	-	-
	Ethynylbenzene	226 304 315 319 335 412	3	0
	Terephthalic acid	315 319 335	1	0
	Benzene	225 304 315 319 340 350 372 412	3	0
Microbiological degradation	Terephthalic acid	315 319 335	1	0

*NFPA:F: Risk of flammability.
**NFPA:R: Risk of reactivity.

Properties related to chemical hazards of the substances involved in PET valorization are presented in **Table S5**. The information was retrieved from the PubChem repository of the National Institute of Health [17] and from the Safety Data Sheets (SDS) found in Sigma-Aldrich® catalogue [18].

Table S6. Price per kilogram of the substances involved in the selected alternatives for chemical valorization of PET, according to United States market.

Alternative	Substance	Price (USD/kg)	Reference
Acid hydrolysis	Terephthalic acid	1.08	[19]
	Ethylene glycol	0.53	[19]
	Sulfuric acid	0.14	[19]
Basic hydrolysis	Terephthalic acid	1.08	[19]
	Ethylene glycol	0.53	[19]
	Sodium hydroxide	0.32	[19]
	Sulfuric acid	0.14	[19]
	Sodium sulphate	0.27	[19]
Glycolysis	Ethylene glycol	0.53	[19]
	Bis(2-hydroxyethyl) terephthalate*	1.00	[20]
	Zinc acetate*	1.36	[21]
Methanolysis	Methanol	0.35	[19]
	Dimethyl terephthalate*	1.09	[22]
	Ethylene glycol	0.53	[19]
	Zinc acetate*	1.36	[21]
	Dichloromethane*	0.57	[22]
Microbiological degradation	Terephthalic acid	1.08	[19]
	Microbiological strain**	278.79	[18]

*Estimated as the average value of reported prices by high profile sellers.
**Value of selling price of 250 mg of product "Lipase acrylic resin" of Merck Millipore®.

Prices of the substances considered in the alternatives are presented in **Table S6**. For the "Incineration" alternative the economic estimations presented in Gradus et al. [23] were used for the calculation of the cost and the revenue value. In the same way, for "Pyrolysis", the estimated cost and revenue were retrieved from the study of Lux [24]. In both cases those values were updated at their present value.

Table S7. Technological Readiness Level (TRL) of the selected alternatives for chemical valorization of PET.

Technology	Readiness	References
Incineration	9	Estimated**
Hydrolysis	3-6	[25]
Glycolysis	3-9	[25]
Methanolysis	6-8	[25]
Pyrolysis*	4-9	[6]
Microbiological degradation	4-6	[26]

*Reported TRL of four (4), typically for the one assisted with plasma or microwaves. TRL of nine (9) for the conventional.
**A value of nine (9) assigned, as is a currently used technology at industrial scales.

Table S8. Indicators matrix from raw data used for sustainability assessment.

		Alternatives						
**Dimension**	**Indicator**	Incineration	Acid hydrolysis	Basic hydrolysis	Glycolysis	Methanolysis	Pyrolysis	Microbiological Degradation
Environmental	ADP (kg Sb eq.)	1.88 E-05	0.24	1.72	3.38 E-03	3.69 E-03	0.00	0.00
	EP (kg PO_4^{3-} eq.)	9.45 E-03	9.50	9.50	43.58	301.66	34.51	24.06
	GWP	1400	5788	5788	76914	437382	25717	0.00
	POFP	0.00	31.26	31.26	415.34	1721.98	138.87	0.00
	TEP (kg PDCB eq.)	0.56	0.00	0.00	0.00	1416.73	0.09	0.00
	HAL	7.75	0.00	0.00	2.00	2.50	2.00	0.00
Economical	Cost (USD)	157	286	2,444	603	7,114	546	279
	Revenue (USD)	352	287	6,040	343	1,271	721	297
Social	Car	1.00	0.00	0.00	0.00	0.50	2.00	0.00
	Mut	0.50	0.00	0.00	0.00	0.50	1.50	0.00
	AT	3.25	0.50	0.50	1.00	2.25	1.00	0.50
	F	1.00	2.00	2.00	3.00	7.00	12.00	1.00
	R	0.00	2.00	3.00	1.00	1.00	2.00	0.00
	RT (°C)	750	180	230	210	230	450	60
	RP (bar)	1	19	19	1	30	1	1
Technological	Maturity	9	6	6	9	8	9	6

Sb: Antimony.
PO_4^{3-}: Phosphate ion.
PDCB: 1,4-Dichlorobenzene.

Figure S1. Comparative figures by indicator of the proposed normalization methods.

Normalization methods are identified as $x = \frac{x_i}{\max x_i}$ (1) , $x = \frac{x_i - \min(x_i)}{\max(x_i) - \min(x_i)}$ (2) , $x = \frac{x_i}{\sum_i x_i}$ (3) , $x = \frac{x_i}{\sqrt{\sum_i {x_i}^2}}$ (4) .

Abiotic Depletion Potential indicator:

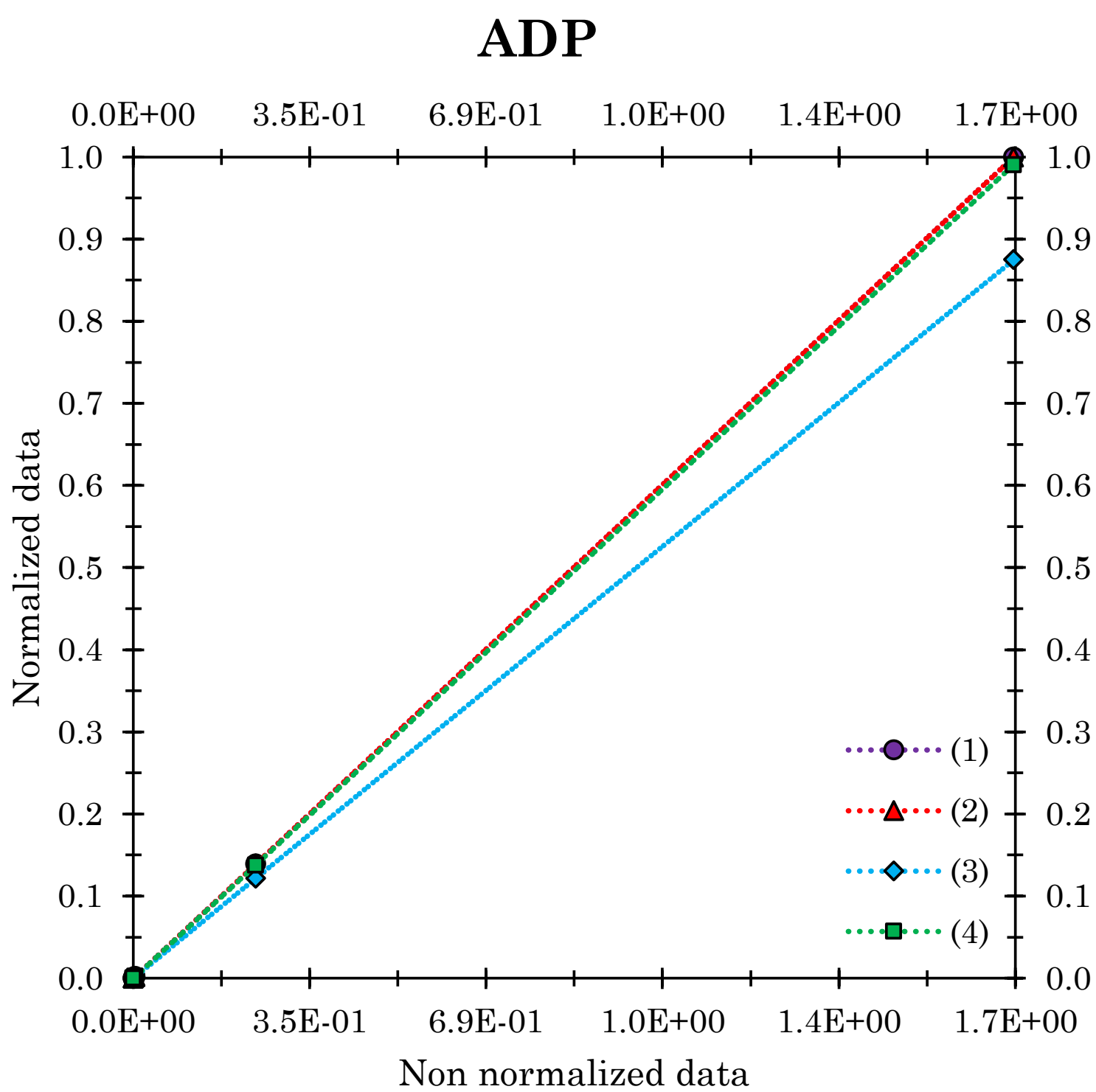

Eutrophication Potential indicator:

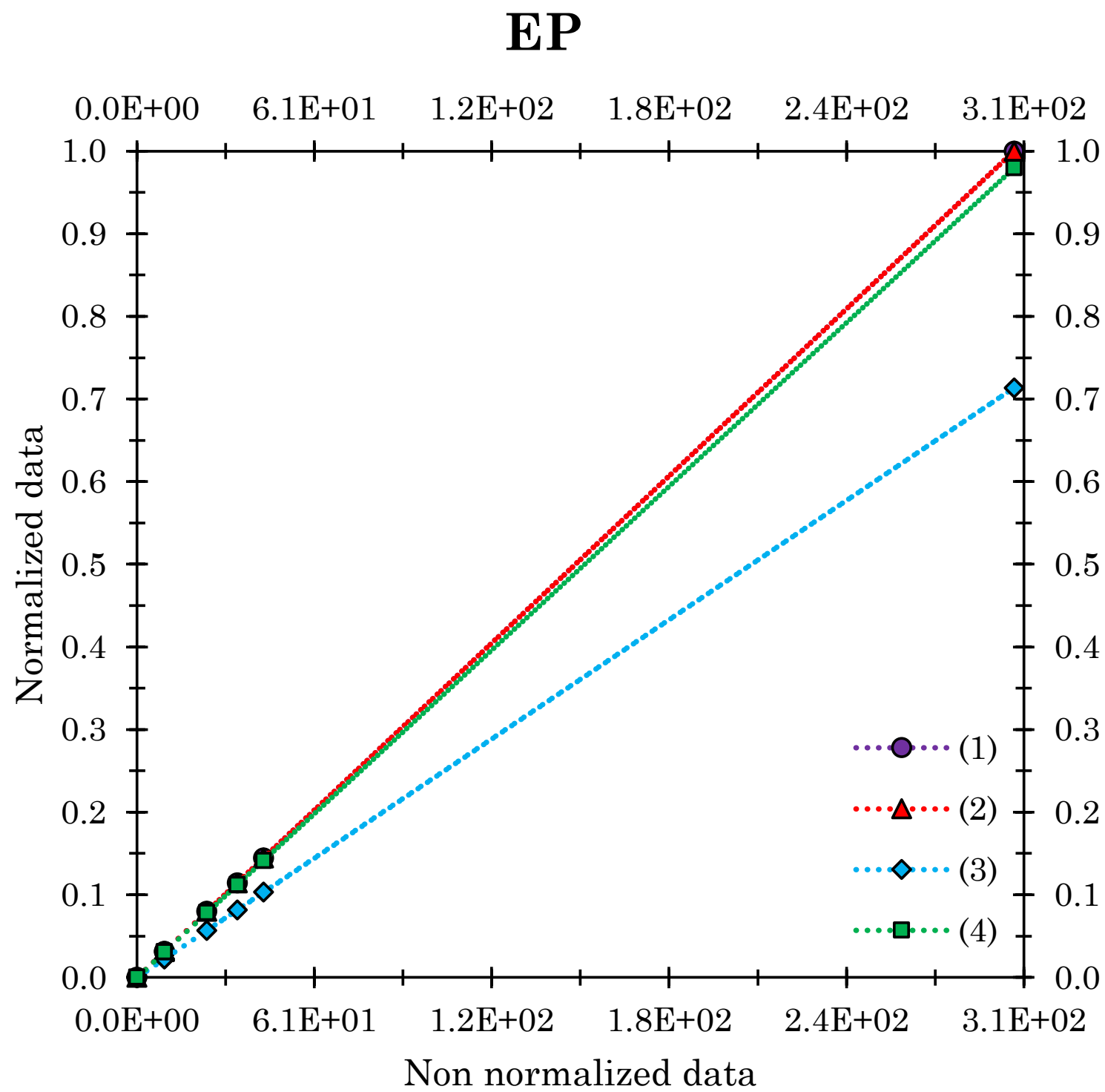

Global Warming Potential indicator:

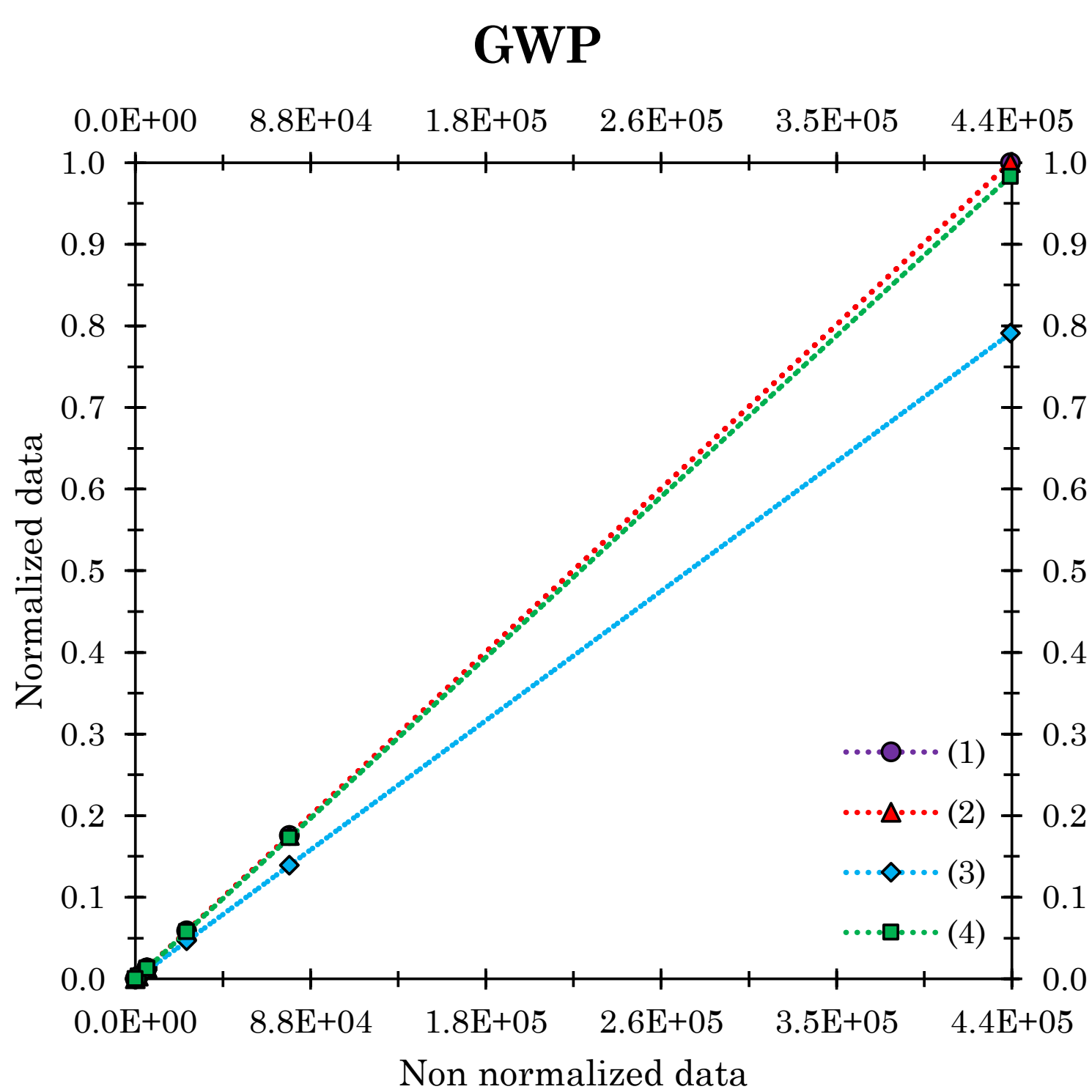

Photochemical Oxidant Formation Potential indicator:

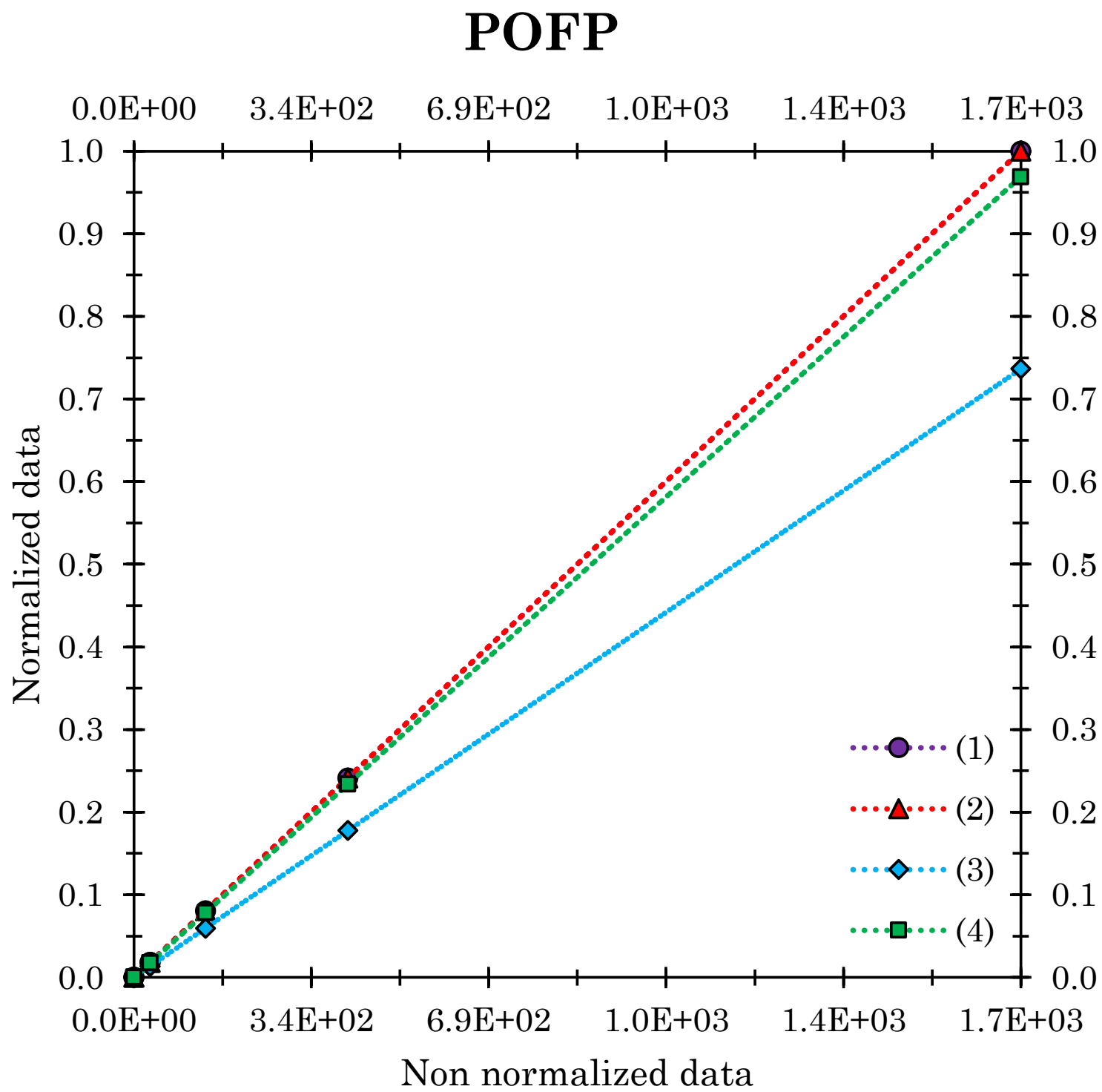

Terrestrial Ecotoxicity Potential indicator:

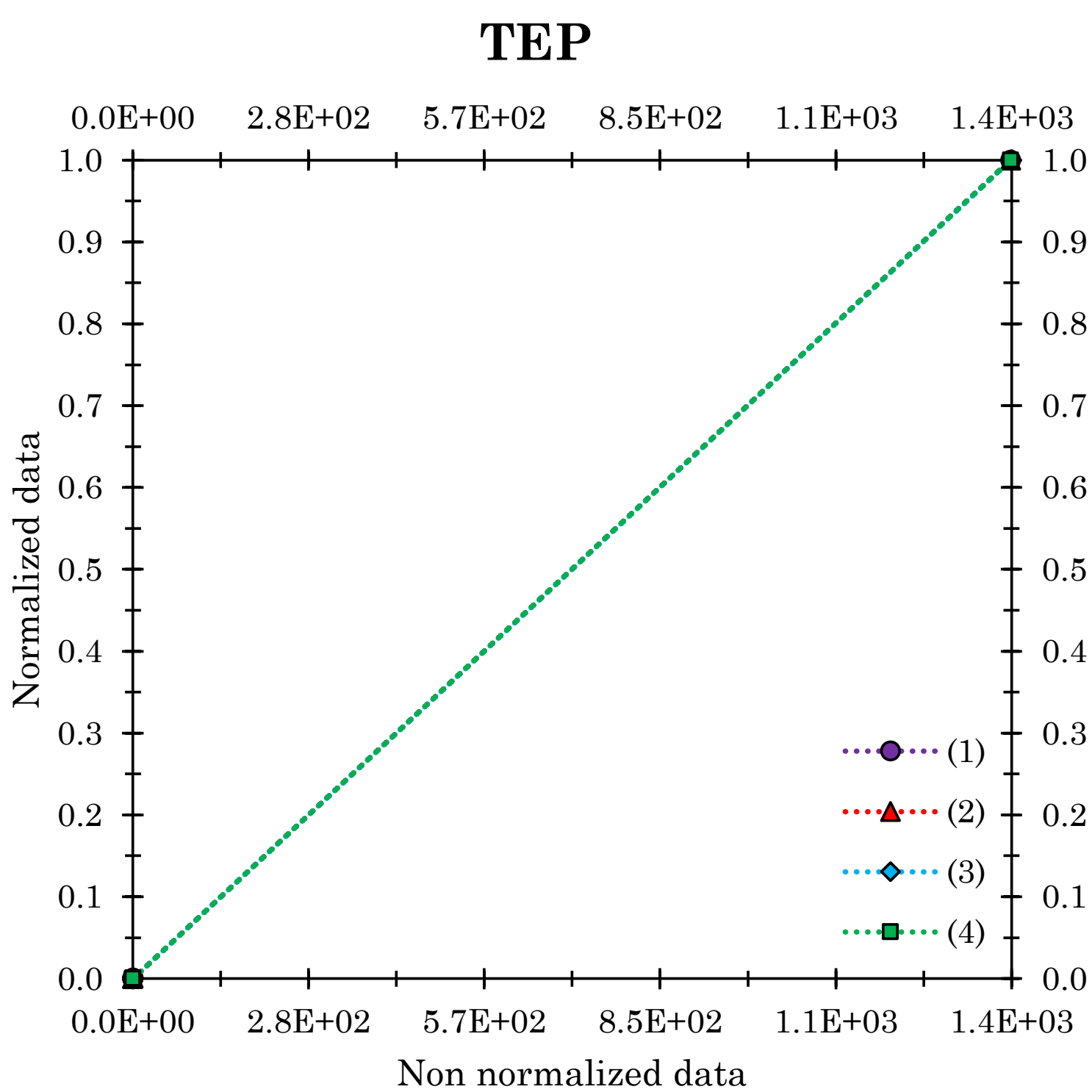

Hazard to Aquatic Life indicator:

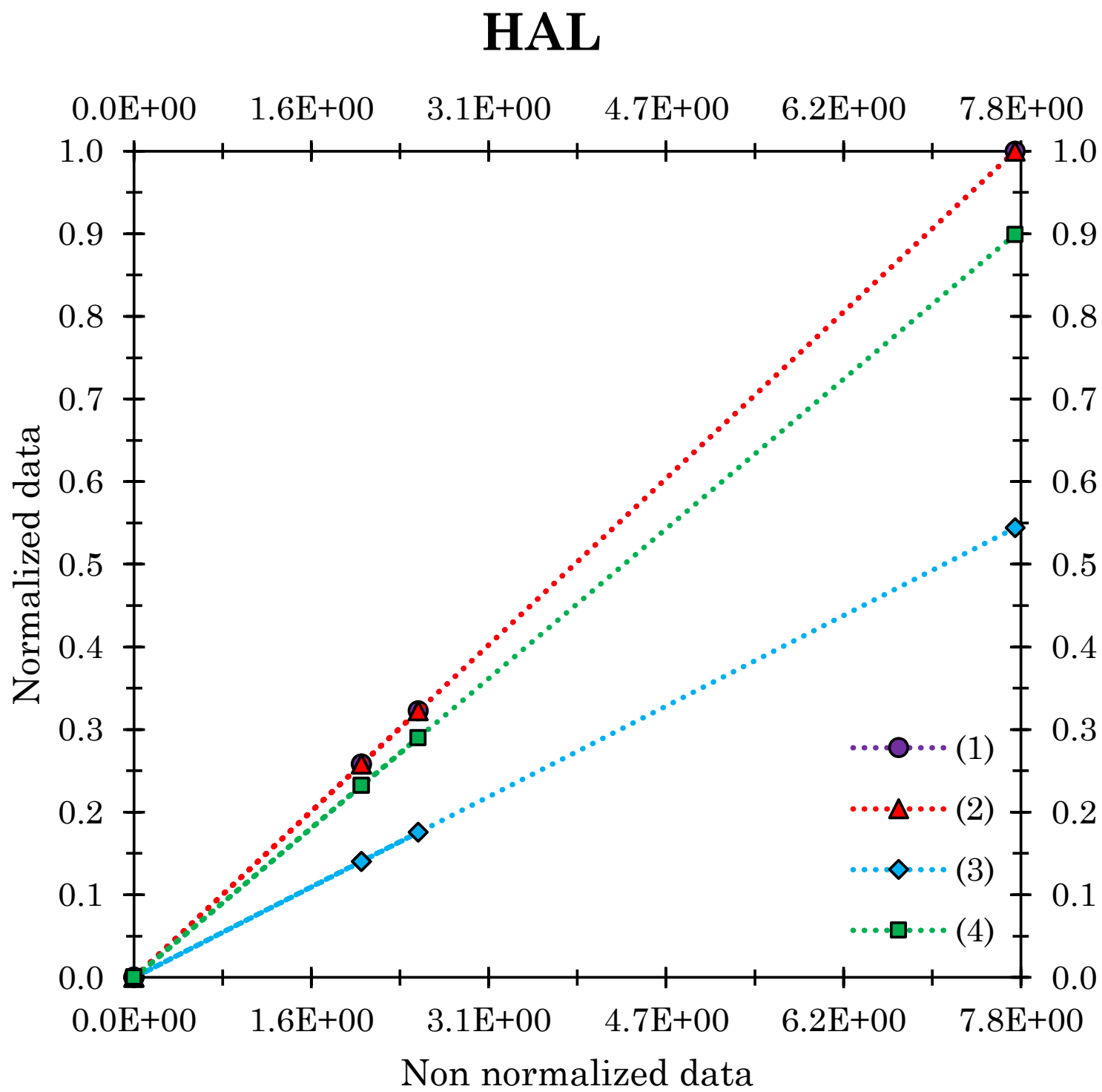

Cost indicator:

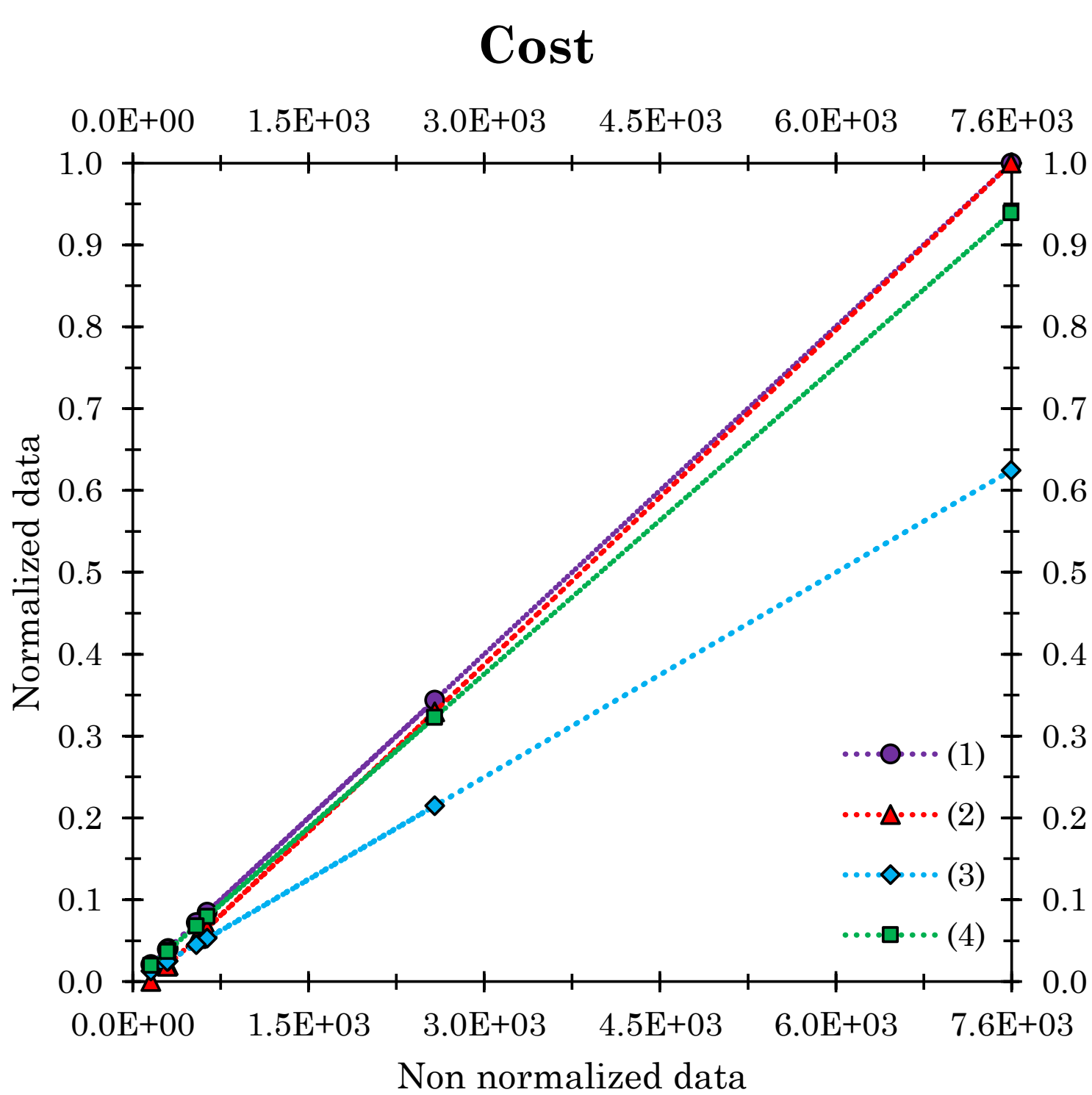

Revenue indicator:

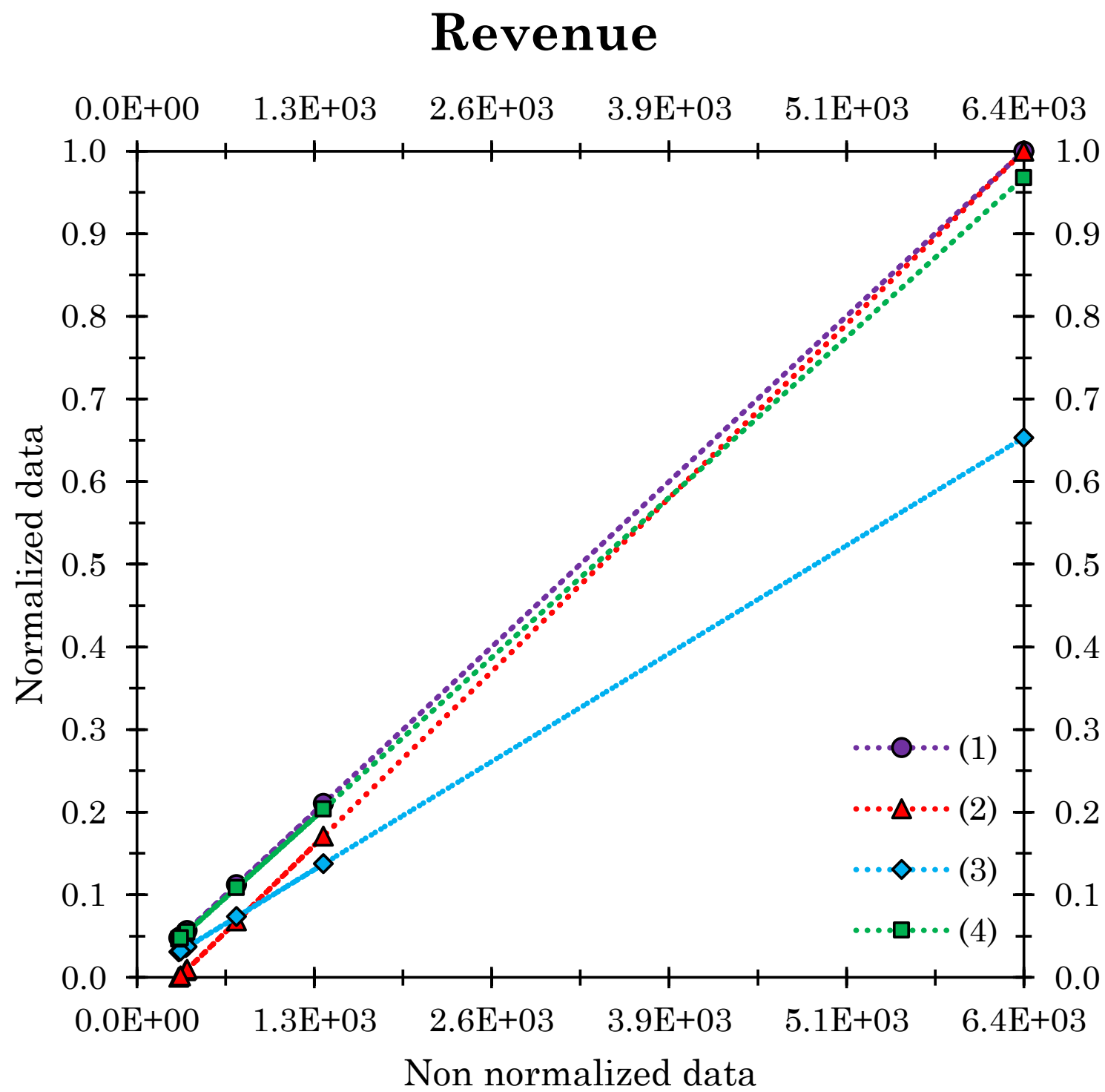

Carcinogenicity indicator:

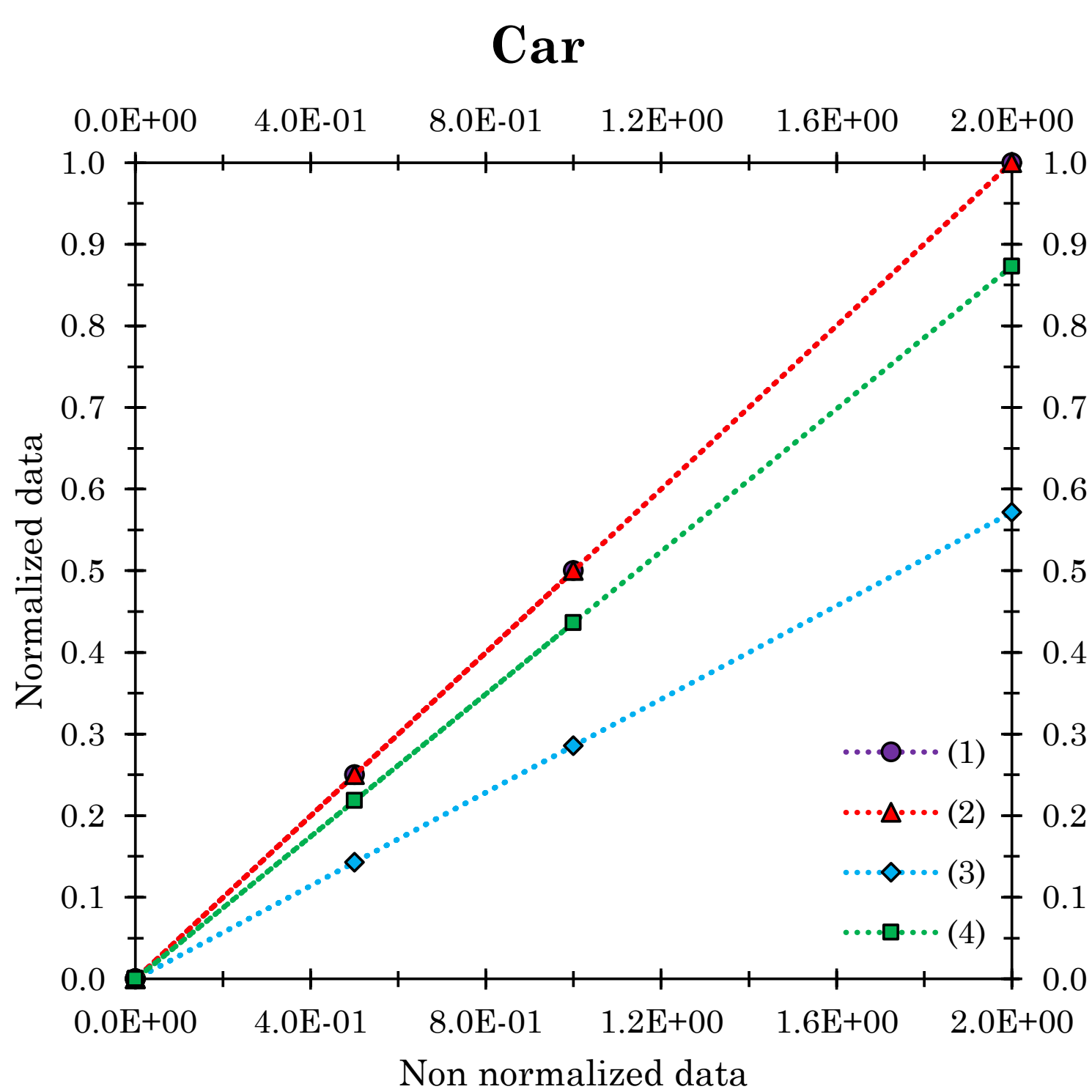

Mutagenicity indicator:

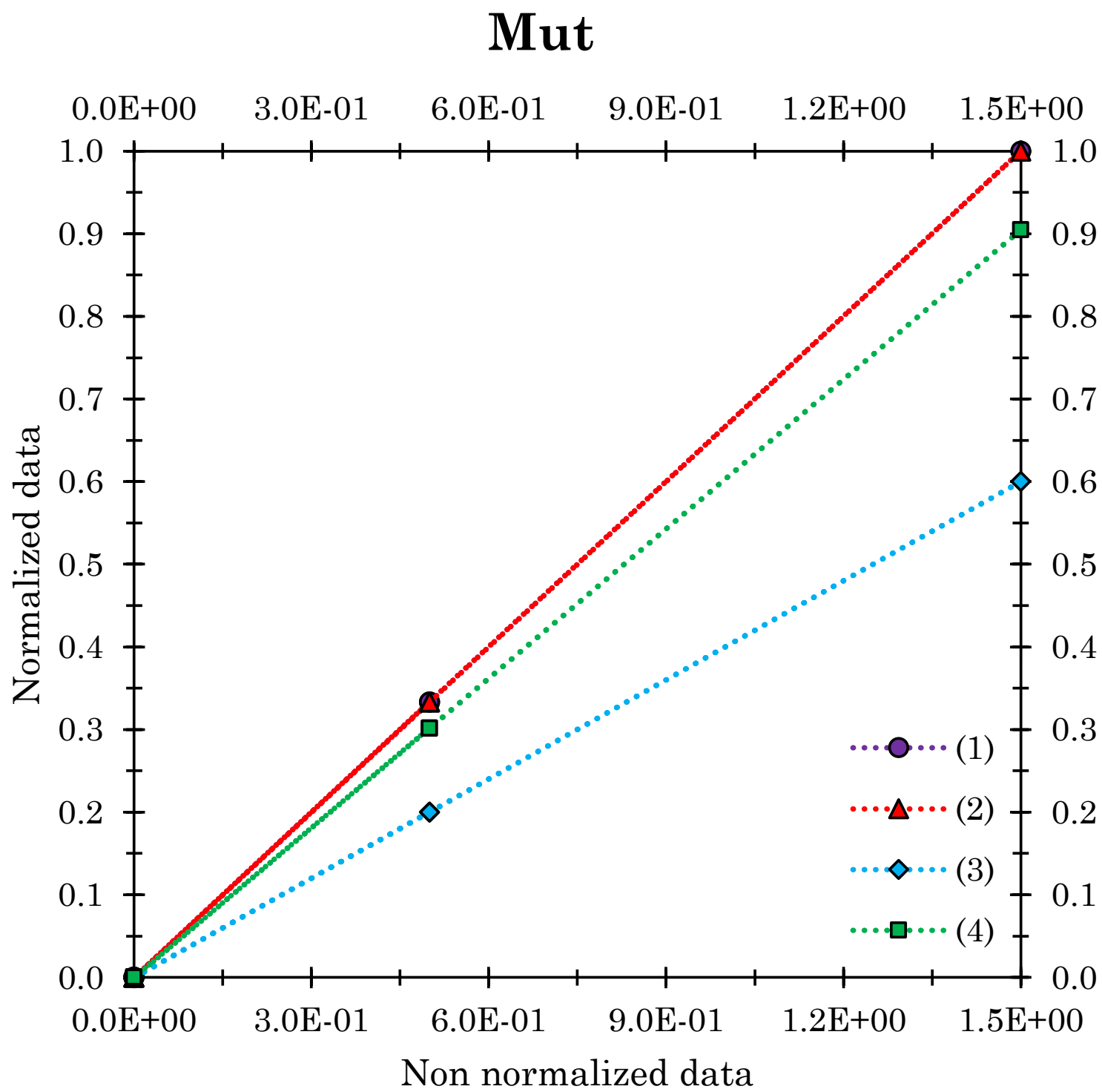

Acute Toxicity indicator:

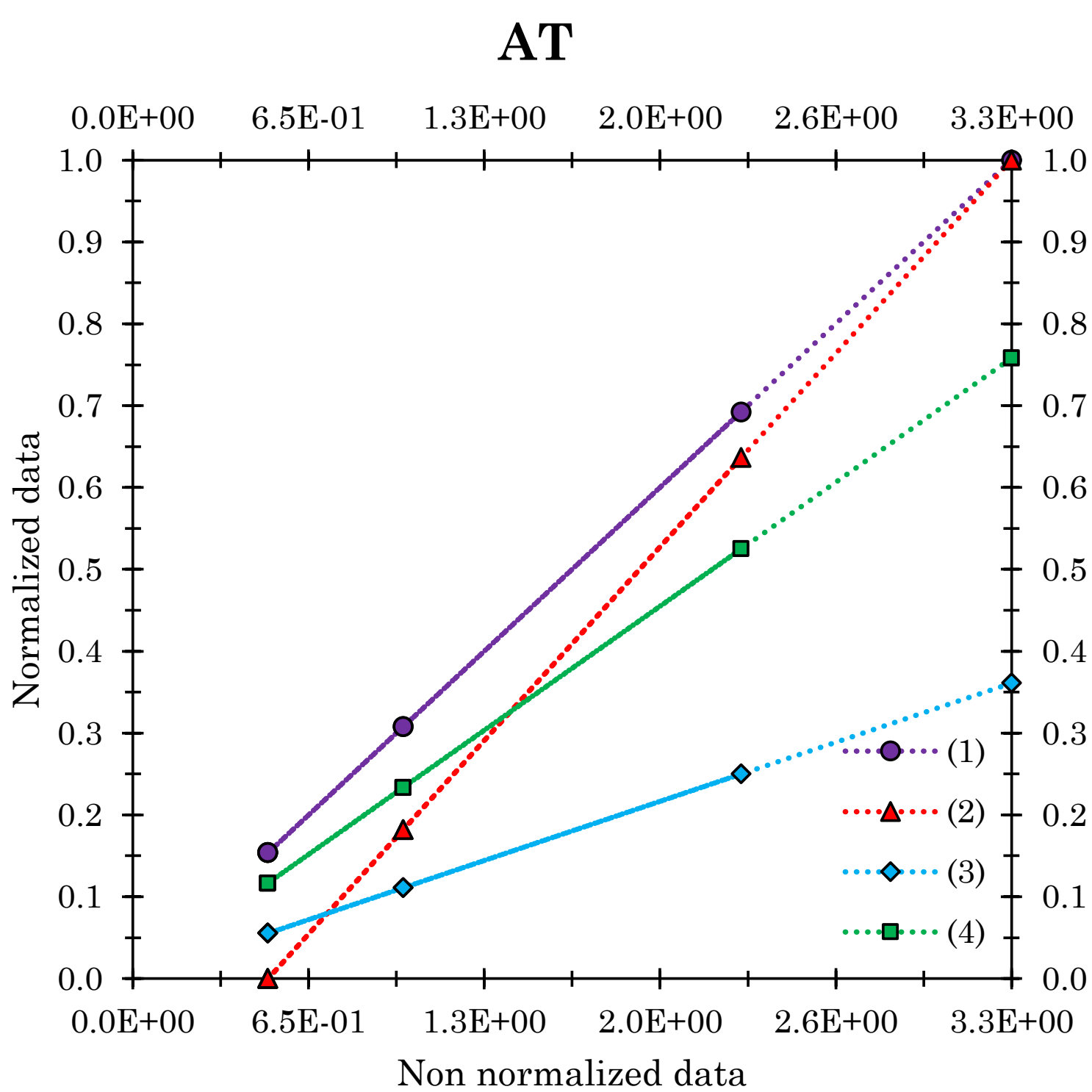

Flammability indicator:

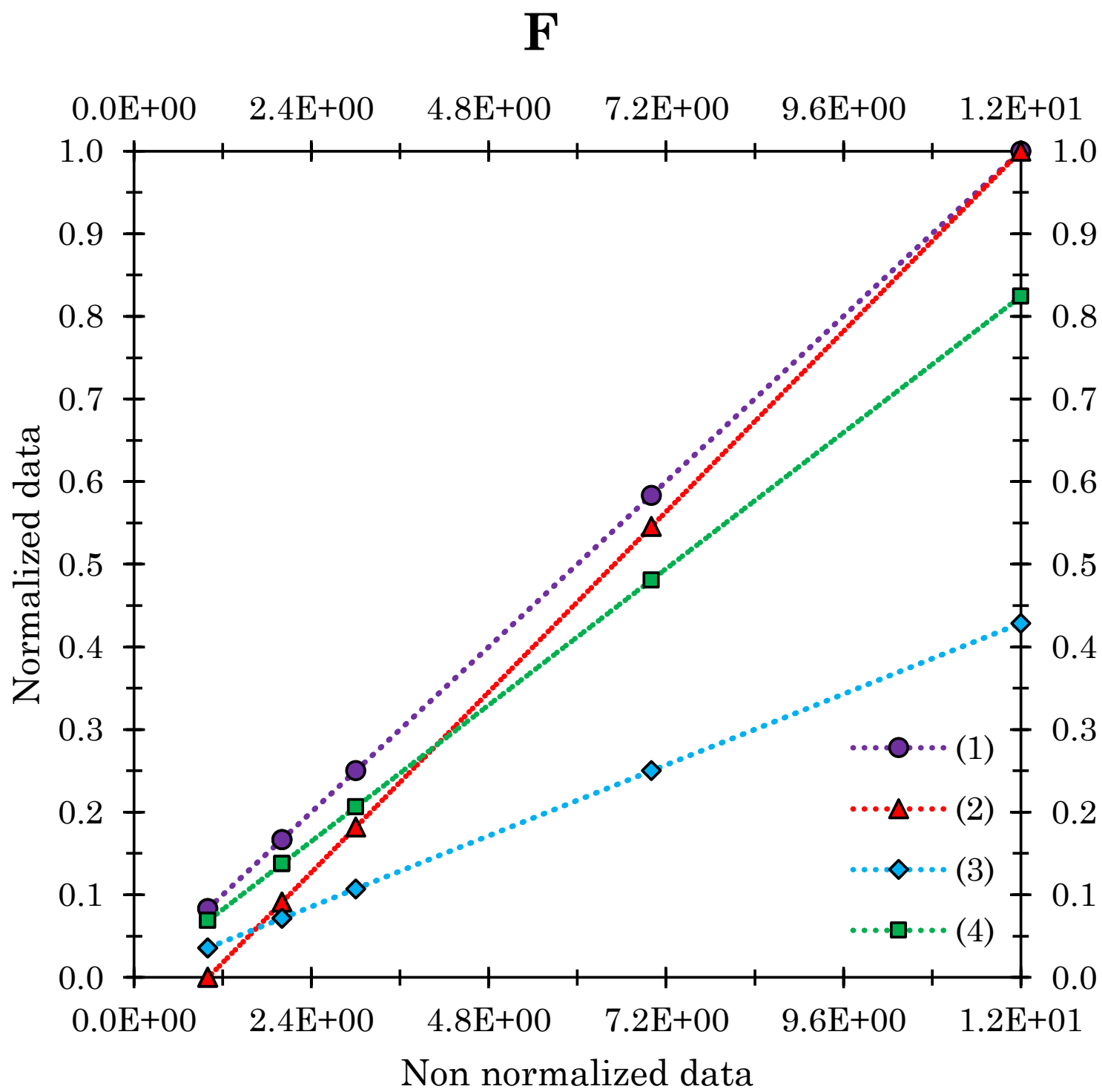

Reactivity indicator:

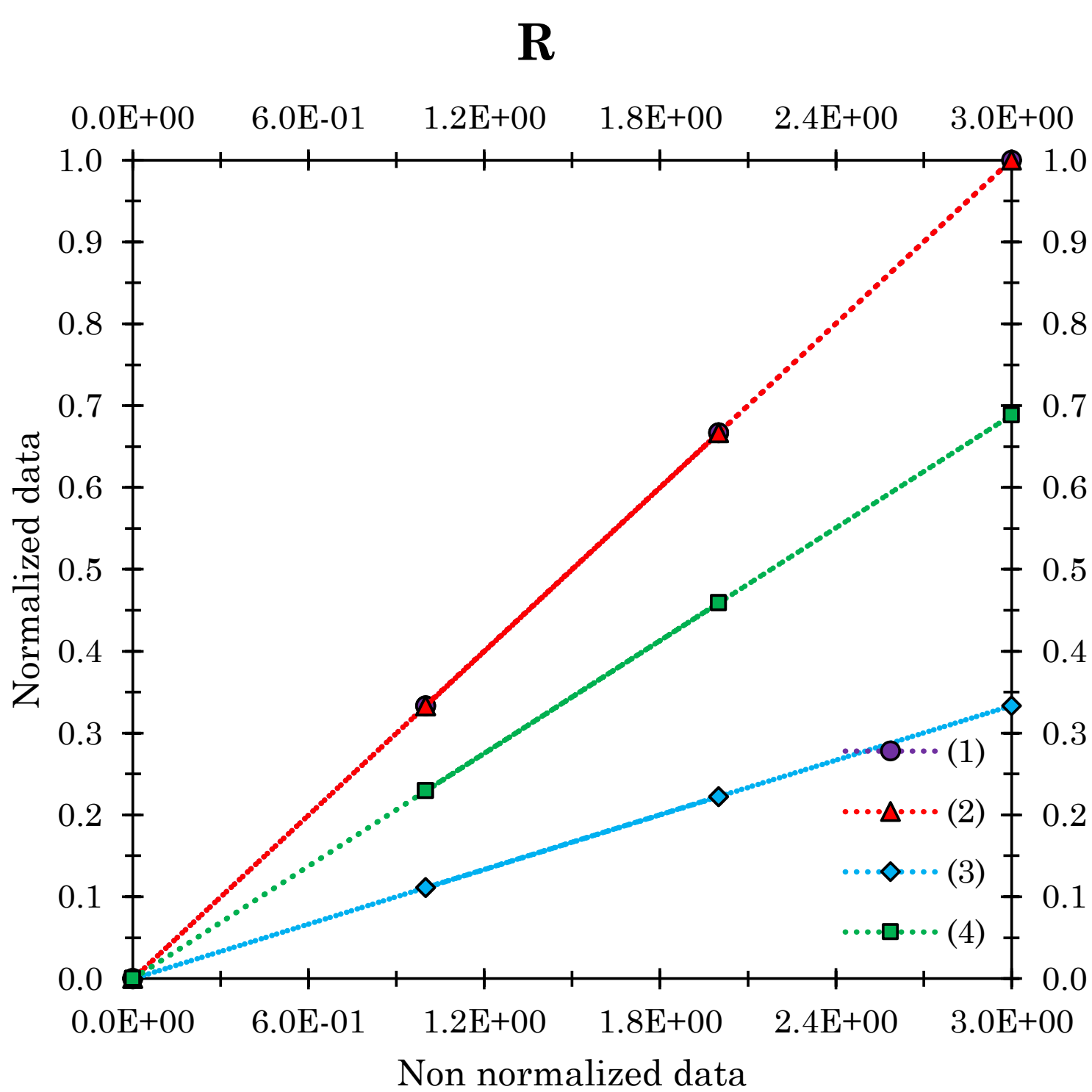

Reaction temperature indicator:

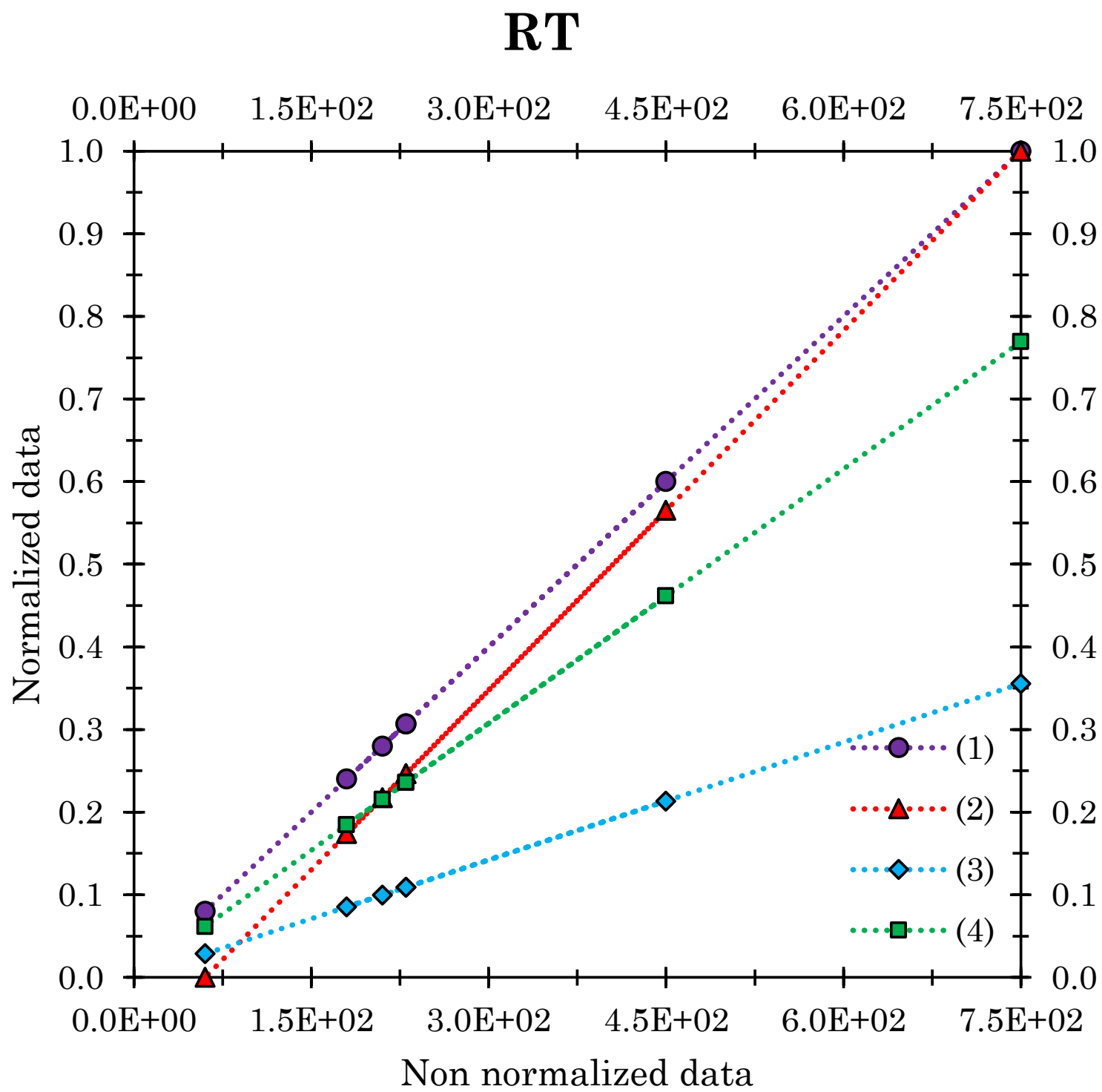

Reaction pressure indicator:

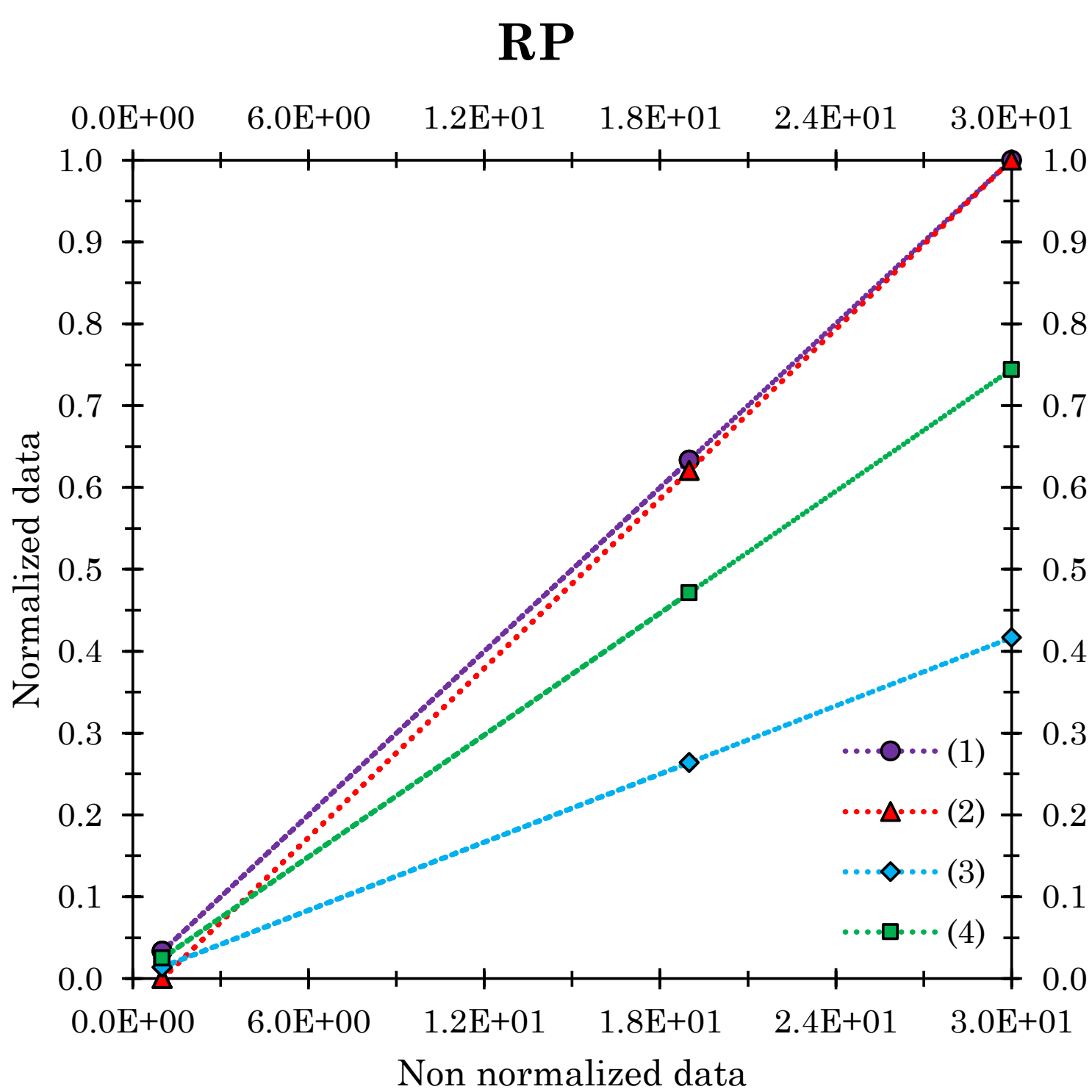

References

[1] A. Amirudin, C. Inoue and G. Grause, "Analyzing Polyethylene Terephthalate Bottle Waste Technology Using an Analytic Hierarchy Process for Developing Countries: A Case Study from Indonesia", *Recycling,* 2022. doi: 10.3390/recycling7040058

[2] The Editors of Encyclopaedia Britannica, "sanitary landfill", January 28 2024. [Online]. Available: https://www.britannica.com/technology/sanitary-landfill.

[3] N. A. Al-Thani, T. Al-Ansari and M. Haouari, "Integrated TOPSIS-COV approach for selecting a sustainable PET waste management technology: A case study in Qatar", *Heliyon,* 2022. doi: 10.1016/j.heliyon.2022.e10274

[4] P. Benyathiar, P. Kumar, G. Carpenter, J. Brace and D. K. Mishra, "Polyethylene Terephthalate (PET) Bottle-to-Bottle Recycling for the Beverage Industry: A Review", *Polymers,* 2022. doi: 10.3390/polym14122366

[5] L. Bartolome, M. Imran, B. Cho, W. Al-Masry and D. Kim, "Recent Developments in the Chemical Recycling of PET", *Material Recycling - Trends and Perspectives*, 2012. doi: 10.5772/33800

[6] M. Solis and S. Silveira, "Technologies for chemical recycling of household plastics – A technical review and TRL assessment", *Waste Management,* vol. 105, pp. 128-138, 2020. doi: 10.1016/j.wasman.2020.01.038.

[7] C. D. Benavides Fernández, M. P. Guzmán Castillo, S. A. Quijano Pérez and L. V. Carvajal Rodríguez, "Microbial degradation of polyethylene terephthalate: a systematic review", *Springer Nature,* 2022. doi: 10.1007/s42452-022-05143-4

[8] R. K. Foolmaun and T. Ramjeeawon, "Comparative life cycle assessment and social life cycle assessment of used polyethylene terephthalate (PET) bottles in Mauritius", *The International Journal of Life Cycle Assessment,* vol. 18, no. 1, 2012. doi: 10.1007/s11367-012-0447-2

[9] T. Chilton, S. Burnley and S. Nesaratnam, "A life cycle assessment of the closed-loop recycling and thermal recovery of post-consumer PET", *Resources, Conservation and Recycling,* vol. 54, 2010. doi: 10.1016/j.resconrec.2010.04.002

[10] G. Carvalho, E. Muniz and A. Rubira, "Hydrolysis of post-consume poly(ethylene terephthalate) with sulfuric acid and product characterization by WAXD, 13C NMR and DSC", *Polymer Degradation and Stability - POLYM DEGRAD STABIL,* vol. 91, no. 6, pp. 1326-1332, 2006. doi: 10.1016/j.polymdegradstab.2005.08.005

[11] A. Aguado, L. Martínez, L. Becerra, M. Arieta-araunabeña, S. Arnaiz, A. Asueta and I. Robertson, "Chemical depolymerisation of PET complex waste: Hydrolysis vs. glycolysis", *Journal of Material Cycles and Waste Management,* vol. 16, no. 2, 2014. doi: 10.1007/s10163-013-0177-y

[12] M. Hofmann, J. Sundermeier, C. Alberti and S. Enthaler, "Zinc(II) acetate Catalyzed Depolymerization of Poly(ethylene terephthalate)", *ChemistrySelect,* vol. 5, no. 32, pp. 10010-10014, 2020. doi: 10.1002/slct.202002260

[13] N. Dimitrov, L. Krehula, A. Ptiček Siročić and Z. Hrnjak-Murgić, "Analysis of recycled PET bottles products by pyrolysis-gas chromatography", *Polymer Degradation and Stability,* vol. 98, pp. 972-979, 2013. doi: 10.1016/j.polymdegradstab.2013.02.013

[14] A. Castro, A. Carniel, J. Junior, A. Gomes and E. Valoni, "Screening of commercial enzymes for poly(ethylene terephthalate) hydrolysis and synergy studies on different substrate sources", *Journal of Industrial Microbiology and Biotechnology 44(5),* vol. 44, 2017. doi: 10.1007/s10295-017-1942-z

[15] C. J. Chirayil, R. K. Mishra and S. Thomas, "Materials Recovery, Direct Reuse and Incineration of PET Bottles", 2019. doi: 10.1016/B978-0-12-811361-5.00003-1

[16] M. Muszyński, J. Nowicki, M. Zygadło and G. Dudek, "Comparsion of Catalyst Effectiveness in Different Chemical Depolymerization Methods of Poly(ethylene terephthalate)", *Molecules,* vol. 28, no. 17, 2023. doi: 10.3390/molecules28176385

[17] National Center for Biotechnology Information, 2024. [Online]. Available: https://pubchem.ncbi.nlm.nih.gov

[18] MilliporeSigma, "Advanced Search", 2024. [Online]. Available: https://www.sigmaaldrich.com/US/en

[19] Business Analytiq, "List of indexes", 2026. [Online]. Available: https://businessanalytiq.com/index/

[20] Guidechem, 2026. [Online]. Available: https://www.guidechem.com/

[21] Made-in-China, 2024. [Online]. Available: https://www.made-in-china.com/

[22] INTRATEC, "Intratec Primary Commodity Prices", 2024. [Online]. Available: https://www.intratec.us/products/primary-commodity-prices.

[23] R. Gradus, R. Koppen, E. Dijkgraaf and P. H. L. Nillesen, "A Cost-Effectiveness Analysis for Incineration or Recycling of Dutch Household Plastics", *SSRN Electronic Journal,* 2016. doi: 10.2139/ssrn.2781933

[24] Lux Research, "The Future of Plastic Recycling", 2019. [Online]. Available: https://luxresearchinc.com/wp-content/uploads/2022/07/lux-research-the-future-of-plastic-recycling-executive-summary.pdf.

[25] E. Harscoet, S. Chouvenc, A. C. Faugeras and F. Ammenti, "Chemical and physico-chemical recycling of plastic waste", 2022. [Online]. Available: https://record-net.org/storage/etudes/21-0919-1A/rapport/Rapport_record21-0919_1A.pdf.

[26] J. C. S. Sales, A. G. Santos, A. M. de Castro and M. A. Z. Coelho, "A critical view on the technology readiness level (TRL) of microbial plastics biodegradation", *World J Microbiol Biotechnol,* vol. 37, no. 7, p. 116, June 14 2021. doi: 10.1007/s11274-021-03089-0
